# *Pectobacterium nicotianae* sp. nov., a novel pathogen causing tobacco hollow stalk disease, is phylogenetically distinct from *P. brasiliense*

**DOI:** 10.3389/fpls.2026.1895702

**Published:** 2026-07-29

**Authors:** Can-Hua Lu, Xiaofang Lu, Houfa Zhou, Jiaen Su, Ning Jiang, Xiao-Tong Gai, Limeng Zhang, Zhen-Yuan Xia, Zhong-Long Lin

**Affiliations:** 1 Yunnan Academy of Tobacco Agricultural Sciences, Kunming, China; 2 College of Tobacco Science, Yunnan Agricultural University, Kunming, China; 3 Lincang City Company, Yunnan Tobacco Company, Lincang, China; 4 Dali Prefecture Company, Yunnan Tobacco Company, Dali, China; 5 China National Tobacco Corporation Yunnan Company, Kunming, China

**Keywords:** hollow stalk of tobacco, *Pectobacterium*, *Pectobacterium nicotianae*, plant-pathogenic bacterium, sp. nov.

## Abstract

Five Gram-negative, facultatively anaerobic bacteria were isolated from the hollow stalk tissues of tobacco plants in Yunnan, China. Based on polyphasic taxonomic data, they were identified as a new *Pectobacterium* species. Phylogenetic analyses of 16S rRNA and the concatenated *dnaX*-*leuS*-*recA* genes grouped them into a distinct monophyletic clade. Genome analyses showed high average nucleotide identity (ANIb 96.96–97.63%) and digital DNA-DNA hybridization (dDDH 76.2–80.1%) values, supporting their classification as a single species. However, three indices, including lower dDDH (67.9%), borderline ANIb (95.89%), and phylogenomic separation with 99% support, differentiate them from *Pectobacterium brasiliense* IPO 3540^T^. Comparative genomics across 24 reference strains revealed differences in virulence factors and specific plant cell wall-degrading enzyme profiles, highlighting adaptations to different hosts. Biochemical tests showed that all isolates lacked β-glucosidase and exhibited unique carbon utilization patterns; chemotaxonomically, strain 21LCBS03^T^ produced only menaquinone MK-8. Pathogenicity experiments confirmed that four strains caused severe soft rot and hypersensitive responses on various hosts, while strain BSHS4, with a dDDH of 76.2% relative to 21LCBS03^T^, showed reduced virulence—possibly a subspecies. Reanalysis of 633 *Pectobacterium* genomes from NCBI reclassified 121 strains into this new species, exposed common misidentifications, and identified 9 candidate novel species awaiting phenotypic validation. Combining phylogenomic, virulence, phenotypic, and pathogenic data, we propose *Pectobacterium nicotianae* sp. nov., with 21LCBS03^T^ (=GDMCC 1.3317^T^ = CCTCC AB2022131^T^ = JCM 35650^T^) designated as the type strain.

## Introduction

The genus *Pectobacterium*, first proposed by Edward L. Waldee in 1945 (Waldee, 1945), belongs to the family *Pectobacteriaceae* within the order *Enterobacterales* (Adeolu et al., 2016). Originally classified within the genus *Erwinia* (Winslow et al., 1920), the genus was redefined in 1998 based on 16S rDNA sequence analysis, leading to the transfer of several *Erwinia* species, including *E. carotovora* subspecies, to *Pectobacterium* (Hauben et al., 1998). Subsequent genome sequencing and phylogenomic analyses have revealed substantial genetic diversity within the genus, resulting in the elevation of five *P. carotovora* subspecies to species rank and the proposal of at least 12 new species over the past two decades (Gardan et al., 2003; Nabhan et al., 2013; Khayi et al., 2016; Dees et al., 2017; Sarfraz et al., 2018; Waleron et al., 2018; Oulghazi et al., 2019; Pédron et al., 2019; Portier et al., 2019; Waleron et al., 2019b; Pasanen et al., 2020; Ben Moussa et al., 2021; Zhou et al., 2022; Hong et al., 2023; Sawada et al., 2024). Currently, *Pectobacterium* comprises 23 validly published species and 5 taxa with preferred names (Meier-Kolthoff et al., 2022).

*Pectobacterium* species are primarily isolated from infected plants and cause soft rot diseases across a wide range of crops, leading to significant pre- and post-harvest agricultural losses (Agnieszka et al., 2022; Valeria et al., 2024). Their pathogenicity mainly results from the production of large amounts of plant cell wall-degrading enzymes (PCWDEs), particularly pectinases, which macerate parenchyma tissues (Gal et al., 2023). Economically important species include *P. carotovorum*, *P. brasiliense* (Felipe et al., 2024), *P. punjabense* (Vasilyeva et al., 2024), *P. polare* (Qin et al., 2024), and *P. versatile* (Handique et al., 2023). *P. carotovorum* has a wide host range, infecting potato (Víchová et al., 2024), pinellia (Luo et al., 2024), kimchi cabbage (Lee et al., 2024), carrot (Imm et al., 2024), and tobacco (Mcintyre et al., 1978; Xia and Mo, 2007). In tobacco, hollow stalk disease is a typical soft rot characterized by blackening and decay at the stem base or top, resulting in hollow stems and wilting leaves. Recent studies in China have reported *Pectobacterium carotovorum* and *Dickeya chrysanthemi* (syn. *Erwinia chrysanthemi*) as causal agents of this disease (Wang et al., 2017; Lin et al., 2020), and *P. versatile* was later isolated from cigar tobacco stems exhibiting similar symptoms (Lu et al., 2023c). Accurate species identification is critical for effective disease management, as different species vary in host range, virulence, and ecological adaptation.

This study reports the isolation and genomic characterization of five *Pectobacterium* strains from tobacco plants with hollow stalk disease in Yunnan Province, China. Comprehensive phylogenetic, genomic, and phenotypic analyses were conducted to determine their taxonomic status. The results support classifying these isolates as a novel species, designated *Pectobacterium nicotianae* sp. nov. Large-scale re-identification of publicly available *Pectobacterium* strains was performed to correct inaccurate species labels in the NCBI database and clarify the taxonomic boundary between *P. nicotianae* and closely related taxa, such as *P. brasiliense*. Furthermore, comparative genomic analysis of 24 *Pectobacterium* strains provided insights into the distribution of secretion systems and PCWDE classes, linking genomic features to ecological adaptation and pathogenic strategies.

## Materials and methods

### Isolation and culture conditions

The tobacco stem or leaf petiole sample from a tobacco plant exhibiting hollow stalk symptoms was surface-sterilized with 70.0% ethanol, rinsed with sterile distilled water three times, and approximately 3–5 cm of tissue from the primarily infected areas was removed. The remaining tissue was cut into small pieces and immersed in 10 mL of sterile distilled water for 10 min, with gentle shaking several times. The resulting cell suspension was subjected to six 10-fold serial dilutions. From each dilution, 100 µL of cell suspension was plated on nutrient agar and incubated at 28 °C for 2 days. Representative single colonies were subcultured twice to obtain pure isolates. Five strains were obtained from two locations in Yunnan Province: 21LCBS03^T^, 21LCBS02, and BSHS4 from tobacco stems of the cultivar (cv.) Yunyan 87 in Lincang, and DL10-2–1 and DL6–4 from tobacco cv. Honghuadajingyuan in a flue-cured barn in Dali (**Table 1**). All strains were stored in 20% glycerol at -80 °C at the Tobacco Plant Protection Laboratory of Yunnan Academy of Tobacco Agricultural Sciences (YATAS), Yuxi, China. Strain 21LCBS03^T^ was deposited with GDMCC 1.3317^T^, CCTCC AB2022131^T^, and JCM 35650^T^.

**Table 1:** Strains used in this study.

Strain	Source	Country	Collected year	16S rDNA	Assembly no.	Reference
21LCBS03^T^ (= GDMCC 1.3317^T^ = CCTCC AB2022131^T^ = JCM 35650^T^)	*Nicotiana tabacum*	China	2021	ON807178	GCA_023272715.1	This study
21LCBS02	*Nicotiana tabacum*	China	2021	PQ559886	GCA_044751965.1	This study
BSHS4	*Nicotiana tabacum*	China	2020	PQ559887	GCA_044751885.1	This study
DL6-4	*Nicotiana tabacum*	China	2020	PQ559888	GCA_044751825.1	This study
DL10-2-1	*Nicotiana tabacum*	China	2020	PQ559889	GCA_044751865.1	This study
*P. actinidiae* KKH3^T^ (= LMG 26003^T^ = KCTC 23131^T^)	*Actinidia chinensis*	South Korea	2006	FJ969376	GCA_000803315.1	(Portier et al., 2019)
*P. aquaticum* A212-S19-A16^T^ (= CFBP 8637^T^ = NCPPB 4640^T^)	River	France	2016	MK035744	GCA_003382565.3	(Oren and Garrity, 2019)
*P. araliae* ICMP 25161^T^ (= MAFF 302110^T^)	*Aralia elata*	Japan	1987	LC773403	GCA_037076465.1	(Sawada et al., 2024)
*P. aroidearum* SCRI 109^T^ (= DSM 27693^T^ = ICMP 1522^T^ = LMG 2417^T^ = NCPPB 929^T^)	*Zantedeschia aethiopica*	South Africa	1959	NR_159926	GCA_041228105.1	(Nabhan et al., 2013)
*P. atrosepticum* CFBP 1526^T^ (= ATCC 33260^T^ = CIP 105192^T^ = DSM 18077^T^ = ICMP 1526^T^ = LMG 2386^T^ = NCPPB 549^T^)	*Solanum tuberosum*	United Kingdom	1957	JN600323	GCA_019056595.1	(Gardan et al., 2003)
*P. betavasculorum* CFBP 1539^T^ (= ATCC 43762^T^ = CFBP 2122^T^ = CIP 105193^T^ = DSM 18076^T^ = ICMP 4226^T^ = LMG 2464^T^ = LMG 2466^T^ = NCPPB 2795^T^)	*Beta vulgaris*	United States of America	1971	JN600328	GCA_000749845.1	(Gardan et al., 2003)
*P. brasiliense* IPO 3540^T^ (= 212^T^ = CFBP 6617^T^ = IBSBF 1692^T^ = NCPPB 4609^T^ = ATCC BAA-417^T^ = LMG 21371^T^)	*Solanum tuberosum*	Brazil	1999	JF926716	GCA_016950255.1	(Portier et al., 2019)
*P. cacticida* CFBP 3628^T^ (= 1-12^T^ = ATCC 49481^T^ = CIP 105191^T^ = DSM 21821^T^ = Dye EH-3^T^ = ICMP 11136^T^ = ICMP 1551-66^T^ = ICPB EC186^T^ = LMG 17936^T^ = NCPPB 3849^T^)	*Carnegiea gigantea*	United States of America	1944	AJ223409	GCA_036885195.1	(Hauben et al., 1998)
*P. carotovorum* CFBP 2046^T^ (= ATCC 15713^T^ = CIP 82.83^T^ = DSM 30168^T^ = HAMBI 1429^T^ = ICMP 5702^T^ = LMG 2404^T^ = NCAIM B.01109^T^ = NCPPB 312^T^ = VKM B-1247^T^)	*Solanum tuberosum*	Denmark	1901	JF926731	GCA_900129615.1	(Skerman et al., 1980)
“*P. colocasium*” LJ1	*Colocasia esculenta*	China	2019	CP084032	GCA_020181655.1	(Zhou et al., 2022)
*P. fontis* M022^T^ (= LMG 30744^T^ = CFBP 8629^T^)	Water	Malaysia	2013	MH627387	GCA_000803215.1	(Oulghazi et al., 2019)
*P. jejuense* 13-115^T^ (= JCM 35940^T^ = KCTC 92800^T^)	*Cucumis sativus*	South Korea	2021	LC742805	GCA_026800125.1	(Hong et al., 2023)
*P. odoriferum* NCPPB 3839^T^ (= CFBP 1878^T^ = CIP 103762^T^ = DSM 22556^T^ = ICMP 11533^T^ = LMG 17566^T^ = LMG 5863^T^)	*Cichorium intybus*	France	1978	AJ223407	GCA_000754765.1	(Portier et al., 2019)
*P. parmentieri* RNS 08-42-1A^T^ (= CFBP 8475^T^ = LMG 29774^T^)	*Solanum tuberosum*	France	2008	CP015749.1	GCA_001742145.1	(Khayi et al., 2016)
*P. parvum* Pcc s0421^T^ (= CFBP 8630^T^ = LMG 30828^T^)	*Solanum tuberosum*	Finland	2004	OANP03000055.1	GCA_900195285.2	(Pasanen et al., 2020)
*P. peruviense* IFB 5232^T^ (= LMG 30269^T^ = PCM 2893^T^ = SCRI 179^T^)	*Solanum tuberosum*	Peru	1979	MF589613	GCA_002847345.1	(Waleron et al., 2018)
*P. polare* NIBIO 1006^T^ (= DSM 105255^T^ = NCPPB 4611^T^)	*Solanum tuberosum*	Norway	2010	MG022083	GCA_040225135.1	(Dees et al., 2017)
*P. polonicum* DPMP 315^T^ (= LMG 31077^T^ = PCM 3006^T^)	Field water	Poland	2016	MK240326	GCA_005497185.1	(Waleron et al., 2019b)
*P. punjabense* SS95^T^ (= CFBP 8604^T^ = LMG 30622^T^)	*Solanum tuberosum*	Pakistan	2017	MH249622	GCA_012427845.1	(Sarfraz et al., 2018)
*P. quasiaquaticum* A477-S1-J17^T^ (= LMG 32181^T^ = CFBP 8805^T^)	River	France	2017	CP065177	GCA_014946775.2	(Ben Moussa et al., 2021)
*P. versatile* CFBP 6051^T^ (= ICMP 9168^T^ = NCPPB 3387^T^)	*Solanum tuberosum*	Netherlands	1975	MK226529	GCA_004296685.1	(Portier et al., 2019)
*P. wasabiae* SR91^T^ (= CFBP 3304^T^ = ATCC 43316^T^ = CIP 105194^T^ = DSM 18074^T^ = ICMP 9121^T^ = LMG 8444^T^ = NCPPB 3701^T^ = PDDCC 9121^T^)	*Eutrema wasabi*	Japan	1985	EF530558	GCA_001742185.1	(Gardan et al., 2003)
“*P. zantedeschiae*” 9M (= DSM 105717 = IFB 9009 = PCM 2892)	*Zantedeschia* sp.	Poland	2005	MG761828	GCA_004137795.1	(Waleron et al., 2019a)
*Dickeya solani* IPO 2222^T^ (= LMG 25993^T^ = NCPPB 4479^T^)	*Solanum tuberosum*	Netherlands	2007	KF639914	GCA_001644705.1	(van der Wolf et al., 2014)

### Pathogenicity tests

Pathogenicity was evaluated on tobacco leaves, stems, and top-removed seedlings, as well as on carrot and potato slices and Chinese cabbage leaves. Single colonies from -80 °C stocks were grown in LB broth at 28 °C for 48 hours, then diluted 10-fold. For tobacco, 100 μL of the diluted culture (~1×10^8^ cfu/mL) was injected into the leaves or stems of 60-day-old plants, which were then incubated at 28 °C for 48 hours in partially sealed plastic bags. For top-removed plants, 50 μL of culture was applied to the cut surface and incubated for 24 hours. Potato and carrot slices (1 cm thick) were inoculated with 10 μL of culture and incubated at 28 °C for 48 hours. For Chinese cabbage, 100 μL of culture was injected into the stalk, and leaves were incubated at 28 °C for 24 hours. Lesion areas were photographed under standardized lighting and measured with ImageJ 1.53s (Schneider et al., 2012), with at least three independent replicates. Sterile distilled water served as the negative control. Statistical significance was assessed by one-way ANOVA (*p* < 0.05) using IBM SPSS Statistics.

### Physiological and biochemical characterization

Five tobacco strains (21LCBS03^T^, 21LCBS02, BSHS4, DL6-4, and DL10-2-1) and four closely related type strains, including *P. brasiliense* LMG 21371^T^ (= IPO 3540^T^) (Portier et al., 2019), *P. quasiaquaticum* LMG 32181^T^ (= A477-S1-J17^T^) (Ben Moussa et al., 2021), *P. polare* DSM 105255^T^ (= NIBIO 1006^T^) (Dees et al., 2017), and *P. parvum* LMG 30828^T^ (= s0421^T^) were compared for phenotypic and chemotaxonomic features using previously described methods (Lu et al., 2022, Lu et al., 2023a). Growth was tested on Luria-Bertani agar (LB), nutrient agar (NA), trypticase soy agar with 5% sheep blood (TSBA), potato glucose agar (PDA), casamino acid peptone glucose (CPG) agar, and Kelman’s tetrazolium chloride agar (TZC) (Kelman, 1954) at 28 °C for 5 days. Cell morphology was examined by transmission electron microscopy (HT 7700; Hitachi) (Liu et al., 2023). Motility was tested on semi-solid medium (SMM) with 0.35% agar (Kelman and Hruschka, 1973). Gram staining was performed with a Gram Stain Kit (D008-1-1; Nanjing Jiancheng Institute of Bioengineering). Growth temperature (10–45 °C), pH (4–10), and NaCl tolerance (0–7%) were assessed (Lu et al., 2023b). Antibiotic susceptibility was tested against 11 antibiotics at standard concentrations, including ampicillin (100 µg/mL), bacitracin (50 µg/mL), chloramphenicol (30 µg/mL), ciprofloxacin (1 µg/mL), cycloheximide (50 µg/mL), gentamicin (100 µg/mL), kanamycin (50 µg/mL), nalidixic acid (30 µg/mL), polymyxin (50 µg/mL), rifamycin (25 µg/mL), and tetracycline (15 µg/mL). Oxidase activity was detected using an oxidase kit (BioMérieux) (Tarrand and Gröschel, 1982), and catalase activity was determined by adding 3.0% H_2_O_2_ to colonies (Clarke and Cowan, 1952). Carbon source utilization was assessed with GEN III MicroPlates (Biolog) (Bochner, 1989). Enzyme activities were measured using API 20NE and API ZYM kits (BioMérieux) (Humble et al., 1977; Holmes et al., 1978). For fatty acid analysis, strains were cultured on TSBA for 48 hours at 28 °C (Sasser, 1990). Lipids and isoprenoid quinones were extracted and analyzed as described (Minnikin and Abdolrahimzadeh, 1971; Collins and Jones, 1981).

### Genome sequencing, assembly, and annotation

Genomic DNA was extracted and sequenced on the DNBSEQ and PacBio platforms. PacBio RSII subreads shorter than 1000 bp were removed. Reads with >50% low-quality bases (≤12) or adapter contamination were filtered. K-mer analysis was used to estimate the genome size prior to assembly. Assembly was performed with Canu v1.5 (Koren et al., 2017) and Falcon v0.3.0 (Chin et al., 2016); single-base errors were corrected with GATK v1.6-13 (Mckenna et al., 2010). GC-depth analysis was used to assess GC content and potential contamination. Genome completeness was assessed with CheckM2 v1.1.0 (Chklovski et al., 2023). Annotation was performed with Bakta v1.11.4 using the full v6 database (Schwengers et al., 2021).

### Phylogenetic and genomic analyses

16S rDNA sequences of all *Pectobacterium* type strains were retrieved from GenBank (Sayers et al., 2025). Sequences were aligned with MAFFT (Katoh and Standley, 2013) and trimmed with Unipro UGENE v51.0 (Okonechnikov et al., 2012). Maximum likelihood (ML), neighbor-joining (NJ), and maximum parsimony (MP) trees were constructed with MEGA v11.0.13 using 1000 bootstrap replicates (Tamura et al., 2021). For multilocus sequence analysis (MLSA), *leuS*, *recA*, and *dnaX* sequences were extracted from genome data, aligned with Clustal Omega (Sievers and Higgins, 2021), and concatenated. Phylogenetic trees were constructed with IQ-TREE v2 using the SH-aLRT test and ultrafast bootstrap with 1000 replicates (Minh et al., 2020); the best-fit model was GTR+F+I+R3.

Average nucleotide identity (ANI) was calculated using the JSpecies WS v4.3.2 web server (Richter et al., 2016) for ANIb (BLAST) and ANIm (MUMmer), and the FastANI script v1.33 (Jain et al., 2018) for FastANI. Digital DNA-DNA hybridization (dDDH) was calculated using the Genome-to-Genome Distance Calculator (GGDC) with recommended formula 2 (dDDH2) (Meier-Kolthoff et al., 2022). Species delineation thresholds were set at dDDH < 70%, ANI < 95–96%, and a monophyletic clade with a bootstrap support value > 70% (Richter and Rosselló-Móra, 2009; Riesco and Trujillo, 2024).

### Comparative genomics of 24 strains

A set of 24 *Pectobacterium* type and representative strains (22 complete and 2 draft genomes) was selected for comparative analysis. Secretion systems were predicted using Macsyfinder v2.1.4 with TXSScan v1.1.3, CONJScan v2.0.1, and TFFscan v1.0.0. Effectors were predicted with BastionHub (Wang et al., 2021) and DeepSecE (Zhang et al., 2023) (minid=50%, mincov=50%). PCWDEs were identified by BLAST against 129 reference proteins from previous studies (Li et al., 2018; Boluk et al., 2021; Zhou et al., 2022; Jonca et al., 2024).

## Results

### Pathogenicity and host range

**Figure 1:**
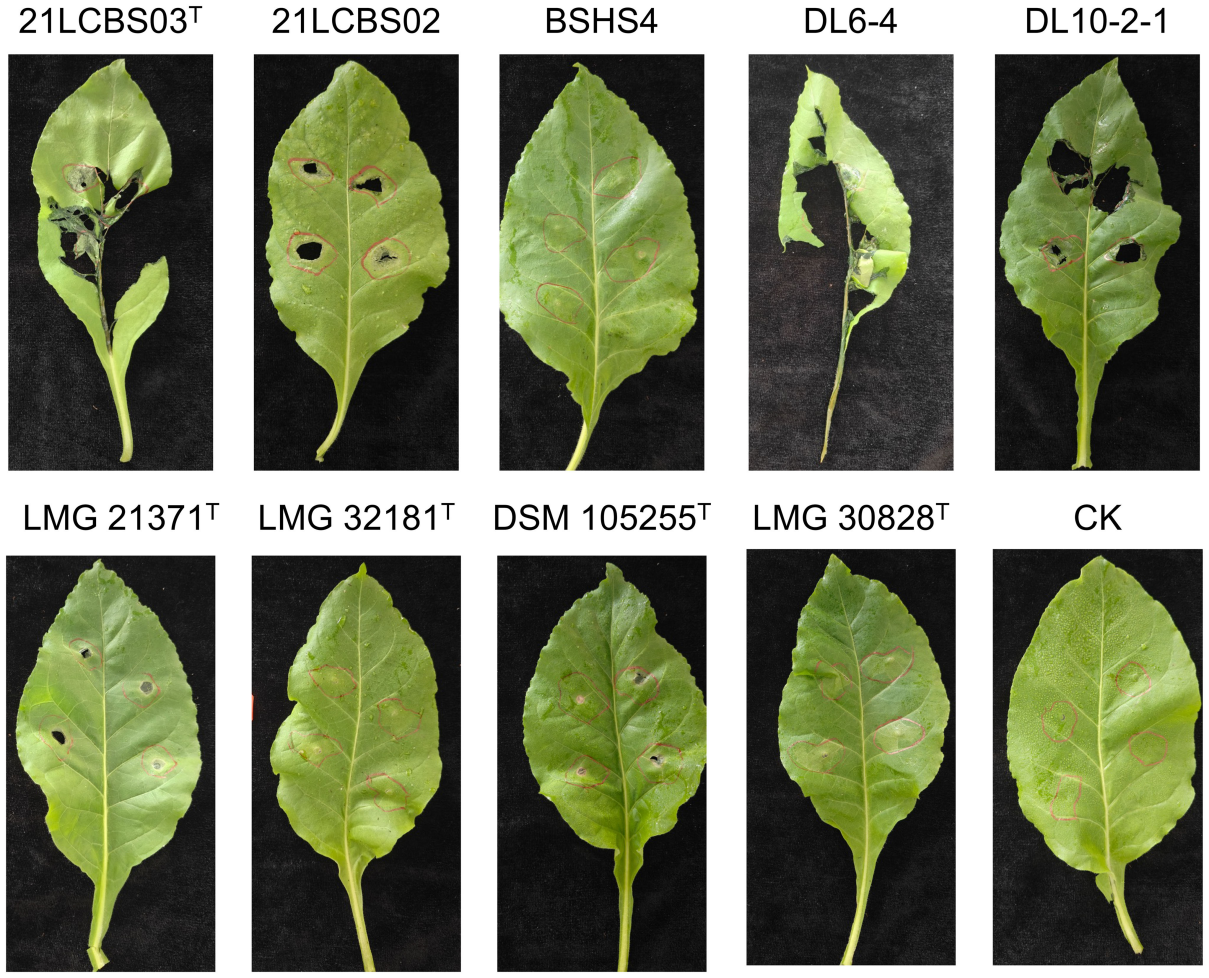
Hypersensitive responses (HR) induced by leaf injection with different *Pectobacterium* strains in *Nicotiana tabacum* cv. Yunyan 87 at 48 hours post-inoculation. All experiments were conducted with three biological replicates, and representative results are shown.

Results showed that four tobacco isolates (21LCBS03^T^, 21LCBS02, DL6-4, and DL10-2-1) induced strong hypersensitive responses (HR) at two days post-inoculation (dpi), whereas strain BSHS4 did not. The *P. brasiliense* LMG 21371^T^ produced smaller lesions. The other three related type strains (*P. quasiaquaticum* LMG 32181^T^ isolated from river water, *P. polare* DSM 105255^T^, and *P. parvum* LMG 30828^T^ isolated from infected potato) did not elicit positive HR responses on tobacco leaves (**Figure 1**). To simulate infection during harvesting, strains were applied to basal and top stem sites. At 24 hours post-inoculation (hpi), most treated seedlings decayed, except those treated with BSHS4 and *P. parvum* LMG 30828^T^, which exhibited significantly lower virulence (**Supplementary Figure 1**). A stem displaying a characteristic symptom from each tobacco isolate inoculation was surface-sterilized, and the bacteria were subsequently reisolated. 16S DNA sequencing confirmed that the recovered isolates were identical to the original inoculated strains, which fully complies with Koch’s postulates. In a separate experiment, soft rot symptoms developed in all tested top-removed seedlings irrigated with tobacco strains, including 21LCBS03^T^, 21LCBS02, DL6-4, and DL10-2-1, at 2 dpi. A similar trend was observed: tobacco isolate BSHS4 and two potato pathogens, *P. polare* DSM 105255^T^ and *P. parvum* LMG 30828^T^, showed significantly lower disease incidence and disease index than the other tested strains (**Supplementary Figure 2**).

The host range was also evaluated using pathogenicity tests on potato, Chinese cabbage, and carrot. All five tobacco isolates caused decay in potato, with the largest lesions produced by 21LCBS03^T^ and DL10-2-1. The closely related potato pathogens *P. brasiliense* LMG 21371^T^, *P. polare* DSM 105255^T^, and *P. parvum* LMG 30828^T^ also caused soft rot in potatoes, while the river water-derived strain *P. quasiaquaticum* LMG 32181^T^ did not (**Supplementary Figure 3**). On carrot slices, all strains except BSHS4 and *P. quasiaquaticum* LMG 32181^T^ resulted in water-soaked lesions; 21LCBS02 and LMG 30828^T^ caused smaller lesions (**Supplementary Figure 4**). On Chinese cabbage, BSHS4, *P. quasiaquaticum* LMG 32181^T^, and *P. polare* DSM 105255^T^ produced smaller lesions, whereas the other four tobacco isolates, along with *P. brasiliense* LMG 21371^T^ and *P. parvum* LMG 30828^T^, exhibited greater virulence (**Supplementary Figure 5**).

The data above suggest that four of five tobacco isolates exhibited strong hypersensitivity and high virulence in tobacco, whereas BSHS4 and some related strains showed lower virulence. All five tobacco isolates caused potato decay, with varying virulence on other hosts and weak or no pathogenicity toward *P. quasiaquaticum* LMG 32181^T^. These differences reflect intraspecific and interspecific pathogenicity diversity across diverse hosts.

### Phylogenetic analysis

**Figure 2:**
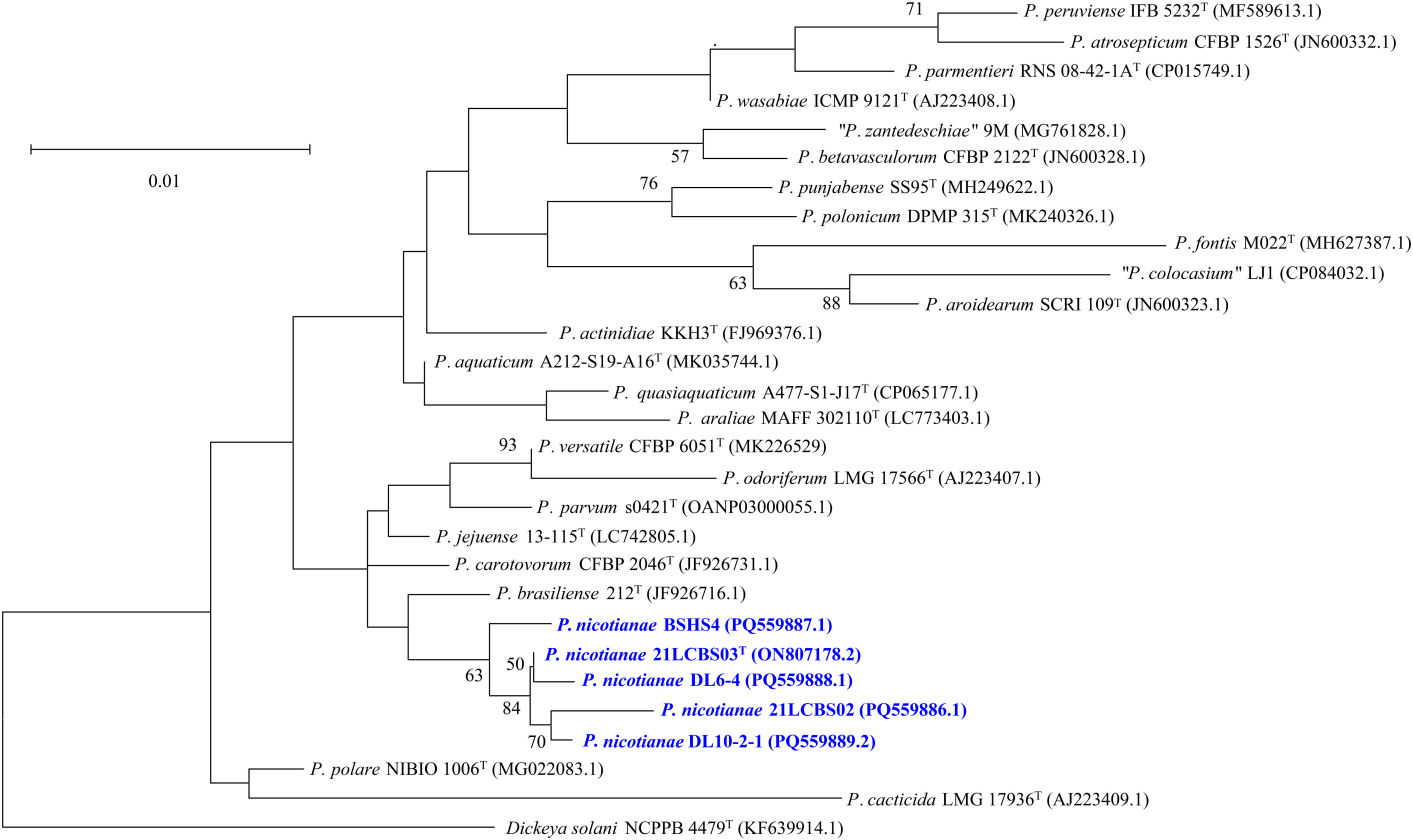
Phylogenetic tree showing the phylogenetic position of five pathogenic strains isolated from the hollow stalk of tobacco among related taxa of the genus *Pectobacterium*, based on partial *16S rRNA* gene sequences (1371 bp). The maximum-likelihood (ML) tree was constructed in MEGA 11 from 1,371 bp of aligned fragments. Bootstrap values (≥50%) from 1,000 replicates are shown at the branch points. Type strains are indicated by superscript T. Sequences obtained in this study are shown in bold blue. Closed circles (●) indicate nodes also recovered in neighbor-joining and maximum-parsimony trees. *Dickeya solani* NCPPB 4479^T^ was used as the outgroup. GenBank accession numbers are in parentheses. Bar, 0.01 substitutions per nucleotide position.

The 16S rDNA analysis (1371 bp) showed that 21LCBS03^T^ shared over 99.50% similarity with the other four tobacco isolates and 99.34% and 99.27% similarity with *P. jejuense* 13-115^T^ and *P. brasiliense* 212^T^, respectively. Other *Pectobacterium* type strains showed 96.12%-99.20% identity to 21LCBS03^T^. These data indicate that the five tobacco strains from hollow stalk disease belong to the genus *Pectobacterium*. Phylogenetic analysis of 16S rDNA sequences in the maximum-likelihood tree (**Figure 2**) showed that the five strains formed a monophyletic clade. They were consistently distinct from *P. brasiliense* 212^T^ and other *Pectobacterium* type strains in neighbor-joining and maximum-parsimony trees (**Supplementary Figures 6** and **7**).

Analysis of the concatenated *dnaX-leuS-recA* sequences (5670 bp) showed that strain 21LCBS03^T^ shared over 97.50% similarity with the other four tobacco isolates and 97.28%, 96.14%, 95.49%, and 95.10% similarity with *P. brasiliense* IPO 3540^T^, *P. jejuense* 13-115^T^, *P. quasiaquaticum* A477-S1-J17^T^, and *P. aquaticum* A212-S19-A16^T^, respectively. Other type strains showed less than 95.00% similarity with 21LCBS03^T^. Sequence similarities among type strains within *Pectobacterium* were below 97.00% (**Supplementary Table 1**). Phylogenetic analysis showed that all five tobacco isolates formed a distinct cluster, supported by 100% values in both SH-aLRT and ultrafast methods. *P. brasiliense* IPO 3540^T^ and *P. jejuense* 13-115^T^ were the closest species to this cluster (**Supplementary Figure 8**). These data suggest that the tobacco strains might represent a novel species within the genus *Pectobacterium*.

### Physiology and chemotaxonomy

Growth variation among the tested strains was evaluated under varying pH, temperature, medium, NaCl tolerance, and antibiotic exposure conditions. For pH tests, all tested strains exhibited robust growth at pH 6–9 but reduced growth at pH 10. The ability of strains to grow at pH 4 or 5 depended on the acid used to adjust pH. For temperature assays, all strains grew well at 15-40 °C but not at 45 °C under aerobic conditions. Strain 21LCBS03^T^ did not grow at 10 °C, whereas other strains showed limited growth. After 5 days at 25 °C, all tobacco strains except BSHS4 grew slightly under anaerobic conditions, whereas *P. brasiliense* LMG 21371^T^, *P. quasiaquaticum* LMG 32181^T^, *P. polare* DSM 105255^T^, and *P. parvum* LMG 30828^T^ did not grow anaerobically. For the media analysis, all isolates and their type strains grew well on the tested media. However, tobacco strains and *P. polare* DSM 105255^T^ grew more vigorously than *P. brasiliense* LMG 21371^T^, *P. quasiaquaticum* LMG 32181^T^, and *P. parvum* LMG 30828^T^. For NaCl tolerance tests, all strains grew well at NaCl concentrations of 0-4% but not at 7%. *P. quasiaquaticum* LMG 32181^T^ and *P. parvum* LMG 30828^T^ were more sensitive and did not grow at 5% NaCl. Strains including 21LCBS03^T^, 21LCBS02, DL10-2-1, and *P. brasiliense* LMG 21371^T^ tolerated 6% NaCl, whereas other strains did not grow at this concentration. Regarding antibiotic resistance, all tested *Pectobacterium* strains were sensitive to ciprofloxacin, gentamicin, polymyxins, and kanamycin, and tolerant to cycloheximide and bacitracin. Most strains tolerated rifamycin at 25 µg/mL. Tobacco strains BSHS4, DL6-4, and DL10-2–1 were tolerant to tetracycline at 1.5 µg/mL, while other strains were sensitive. Notably, BSHS4 grew well in medium with 100 µg/mL ampicillin.

In the API 20NE and API ZYM assays, all nine *Pectobacterium* strains were positive for 12 reactions and negative for 11 reactions, respectively. All strains were catalase-positive and cytochrome oxidase-negative, consistent with previous reports (Pasanen et al., 2020; Hong et al., 2023). In API 20NE assays, four of five tobacco isolates (21LCBS03^T^, BSHS4, DL6-4, and DL10-2-1) produced indole from L-tryptophan. In contrast, 21LCBS02 and the type strains of *P. brasiliense* LMG 21371^T^, *P. quasiaquaticum* LMG 32181^T^, *P. polare* DSM 105255^T^, and *P. parvum* LMG 30828^T^ were negative. *P. polare* DSM 105255^T^ and tobacco strains 21LCBS03^T^, 21LCBS02, and DL6–4 could not assimilate potassium gluconate, whereas BSHS4, DL10-2-1, and the other type strains showed weak or strong utilization. In API ZYM tests, all strains except *P. quasiaquaticum* LMG 32181^T^ were unable to hydrolyze esterase lipase (C8). All five tobacco strains, *P. brasiliense* LMG 21371^T^ and *P. polare* DSM 105255^T,^showed positive α-glucosidase activity*. P. quasiaquaticum* LMG 32181^T^ and *P. parvum* LMG 30828^T^ were negative. The five tobacco isolates, *P. polare* DSM 105255^T^, and *P. parvum* LMG 30828^T^ were negative for β-glucosidase, whereas *P. brasiliense* LMG 21371^T^ and *P. quasiaquaticum* LMG 32181^T^ were positive. Strains 21LCBS03^T^, BSHS4, DL6-4, and DL10-2-1, along with *P. quasiaquaticum* LMG 32181^T^ and *P. polare* DSM 105255^T^, were negative for esterase (C4) and leucine arylamidase, while 21LCBS02, *P. brasiliense* LMG 21371^T^, and *P. parvum* LMG 30828^T^ were positive. These results show that 21LCBS02 shares more enzymatic similarities with *P. brasiliense* LMG 21371^T^ than the other tobacco strains. However, all tobacco strains differ from *P. brasiliense* LMG 21371^T^ in β-glucosidase activity.

In Gen III assays, although all tested strains showed similar patterns across many carbon sources and inhibitory chemicals, some differences were observed. For example, *P. parvum* LMG 30828^T^ did not utilize D-raffinose or citric acid, whereas the five tobacco isolates and other type strains did. Only *P. polare* DSM 105255^T^ grew well in the presence of potassium tellurite. *P. quasiaquaticum* LMG 32181^T^ weakly utilized L-fucose and was negative for D-trehalose, while other strains showed the opposite. *P. brasiliense* LMG 21371^T^ hydrolyzed L-lactic acid and α-hydroxy-butyric acid but did not utilize D-glucuronic acid, unlike the other strains. Most tobacco strains and *P. brasiliense* LMG 21371^T^ grew weakly in the presence of 8% NaCl, minocycline, or aztreonam, whereas other type strains did not. Collectively, these biochemical features support the identification of a novel species within the genus *Pectobacterium*. A summary of these distinguishing phenotypic features is provided in **Table 2**.

**Table 2:** Phenotypic differentiation among tobacco isolates and closely related type strains of the genus *Pectobacterium*.

Characteristics	1	2	3	4	5	6	7	8	9
pH 4 (titrated with nitric acid)	–	–	–	–	–	w	w	–	–
pH 5 (titrated with glycine)	w	w	–	w	–	–	w	–	w
TZC medium	+	+	+	+	+	w	w	+	w
Anaerobic condition	w	w	–	w	w	–	–	–	–
6% NaCl	w	w	–	–	w	+	–	–	–
Antibiotics:									
1.5 µg/mL tetracycline	–	–	w	w	w	–	w	–	–
10 µg/mL chloramphenicol	–	+	w	–	–	–	w	–	–
100 µg/mL ampicillin	–	–	+	–	–	–	–	–	–
10 µg/mL ampicillin	w	w	+	w	w	w	–	w	w
3 µg/mL nalidixic acid	+	+	–	+	w	w	+	w	+
API ZYM:									
Esterase (C4)	–	+	–	–	–	+	+	–	+
Leucine arylamidase	–	+	–	–	–	+	+	–	+
β-glucosidase	–	–	–	–	–	+	+	–	–
Esterase lipase (C8)	–	–	–	–	–	–	+	–	–
α-glucosidase	+	+	+	+	+	+	–	+	–
API 20NE:									
Indole production	+	–	+	+	+	–	–	–	–
Potassium gluconate assimilation	–	–	+	–	w	w	+	–	w
Gen III:									
D-glucuronic acid	w	w	+	w	w	–	w	w	w
L-lactic acid	-	-	–	–	–	+	–	–	-
α-hydroxy-butyric acid	-	-	–	–	–	+	–	–	-
L-alanine	-	-	w	–	–	+	w	–	-
D-lactic acid methyl ester	-	-	–	w	w	+	–	–	-
α-keto-glutaric acid	-	-	–	–	–	w	w	w	-
D-malic acid	-	-	w	–	–	w	w	w	-
D-trehalose	+	+	+	+	+	+	–	+	+
L-fucose	-	-	–	–	–	–	w	–	-
Potassium tellurite	-	-	–	–	–	–	–	+	-
Stachyose	+	+	+	+	+	+	–	+	-
D-raffinose	+	+	+	+	+	+	+	+	-
Citric acid	+	+	+	+	+	+	+	+	-

Strains: 1, 21LCBS03^T^; 2, 21LCBS02; 3, BSHS4; 4, DL6-4; 5, DL10-2-1; 6, *P. brasiliense* LMG 21371^T^; 7, *P. quasiaquaticum* LMG 32181^T^; 8, *P. polare* DSM 105255^T^; 9, *P. parvum* LMG 30828^T^. All data were obtained in this study. Symbols: +, positive; -, negative; w, weak activity.

Gas chromatography analysis showed that all tested *Pectobacterium* strains shared a conserved fatty acid profile, including saturated fatty acids C_12:0_, C_14:0_, C_16:0_, C_17:0_, and C_18:0_; the unsaturated fatty acid C_17:1_ ω8c; and summed features 2 (C_14:0_ 3-OH/C_16:1_ iso I), 3 (C_16:1_ ω7c/C_16:1_ ω6c), and 8 (C_18:1_ ω7c/C_18:1_ ω6c) (**Table 3**). The major fatty acids in 21LCBS03^T^ were C_12:0_ (5.89 ± 0.08%), C_16:0_ (29.03 ± 0.06%), summed feature 2 (8.22 ± 0.24%), summed feature 3 (30.75 ± 0.27%), and summed feature 8 (20.53 ± 0.25%), consistent with closely related *Pectobacterium* type strains (Hong et al., 2023; Sawada et al., 2024). Additionally, 21LCBS03^T^ contained diphosphatidylglycerol, phosphatidylglycerol, phosphatidylethanolamine, one unidentified aminolipid, two unidentified lipids, and three unidentified aminophospholipids (**Supplementary Figure 9**). Only the respiratory menaquinone MK-8 (MK-8) was detected in 21LCBS03^T^ (**Supplementary Figure 10**). However, a previous study identified ubiquinone-8 (Q-8) and MK-8 in seven type strains of *Pectobacterium*, including *P. brasiliense* CFBP 6617^T^ and *P. carotovorum* CFBP 2046^T^ (Nabhan et al., 2013).

**Table 3:** Cellular fatty acid compositions of strain 21LCBS03^T^ and closely related *Pectobacterium* type strains.

Fatty acid	1	2	3	4	5	6	7	8	9
Saturated:									
C_12:0_	5.89 ± 0.08	6.00 ± 0.13	6.31 ± 0.06	6.35 ± 0.07	6.50 ± 0.08	6.24 ± 0.03	6.12 ± 0.53	5.82 ± 0.09	5.72 ± 0.01
C_13:0_	0.23 ± 0.02	TR	0.70 ± 0.01	–	TR	TR	TR	TR	1.00 ± 0.05
C_14:0_	1.25 ± 0.01	1.33 ± 0.03	1.94 ± 0.39	1.85 ± 0.48	1.98 ± 0.43	1.92 ± 0.08	2.13 ± 0.13	1.63 ± 0.54	1.58 ± 0.26
C_16:0_	29.03 ± 0.06	28.32 ± 0.32	25.89 ± 0.20	28.91 ± 0.21	27.79 ± 0.29	29.00 ± 0.16	27.24 ± 1.09	25.85 ± 0.09	24.92 ± 0.65
C_17:0_	1.12 ± 0.03	1.18 ± 0.02	2.09 ± 0.10	1.10 ± 0.07	1.63 ± 0.03	1.56 ± 0.06	0.94 ± 0.07	1.18 ± 0.05	1.89 ± 0.22
C_18:0_	2.19 ± 0.08	1.37 ± 0.04	2.06 ± 0.11	2.28 ± 0.06	2.19 ± 0.03	1.37 ± 0.55	2.82 ± 0.71	2.18 ± 0.15	2.03 ± 1.18
Unsaturated:									
C_17:1_ ω8c	0.40 ± 0.03	0.42 ± 0.01	1.09 ± 0.06	0.68 ± 0.03	0.67 ± 0.04	0.47 ± 0.03	0.34 ± 0.04	0.82 ± 0.01	1.39 ± 0.12
Summed features: ^a^									
1	–	–	–	–	–	–	–	–	0.94 ± 0.06
2	8.22 ± 0.24	8.41 ± 0.32	9.50 ± 0.09	9.61 ± 0.18	9.67 ± 0.13	8.39 ± 0.08	7.83 ± 0.23	9.56 ± 0.16	8.68 ± 0.54
3	30.75 ± 0.27	30.76 ± 0.11	30.25 ± 0.19	31.74 ± 0.20	30.83 ± 0.11	30.70 ± 0.52	34.99 ± 1.04	33.42 ± 0.13	31.99 ± 0.04
8	20.53 ± 0.25	21.51 ± 0.03	20.18 ± 0.20	17.94 ± 0.21	18.31 ± 0.19	19.66 ± 0.32	15.96 ± 0.14	19.25 ± 0.06	17.98 ± 1.07

Strains: 1, 21LCBS03^T^; 2, 21LCBS02; 3, BSHS4; 4, DL6-4; 5, DL10-2-1; 6, *P. brasiliense* LMG 21371^T^; 7, *P. quasiaquaticum* LMG 32181^T^; 8, *P. polare* DSM 105255^T^; 9, *P. parvum* LMG 30828^T^. Data are shown as percentages of total fatty acids. Compounds at 0.5% or higher are listed. TR, trace amount (less than 0.5% of the total); -, not detected.

^a^ Summed Features are fatty acids that cannot be resolved reliably from other fatty acids using the chromatographic conditions chosen. The MIDI system groups these fatty acids into a single feature, with a single percentage of the total. Summed feature 1 comprises C_13:0_ 3-OH/C_15:1_ OH. Summed feature 2 comprises C_14:0_ 3-OH/C_16:1_ iso I. Summed feature 3 comprises C_16:1_ ω7*c* and/or C_16:1_ ω6*c*. Summed feature 8 comprises C_18:1_ ω7*c* and/or C_18:1_ ω6*c*.

### Genome features among tobacco isolates

**Figure 3:**
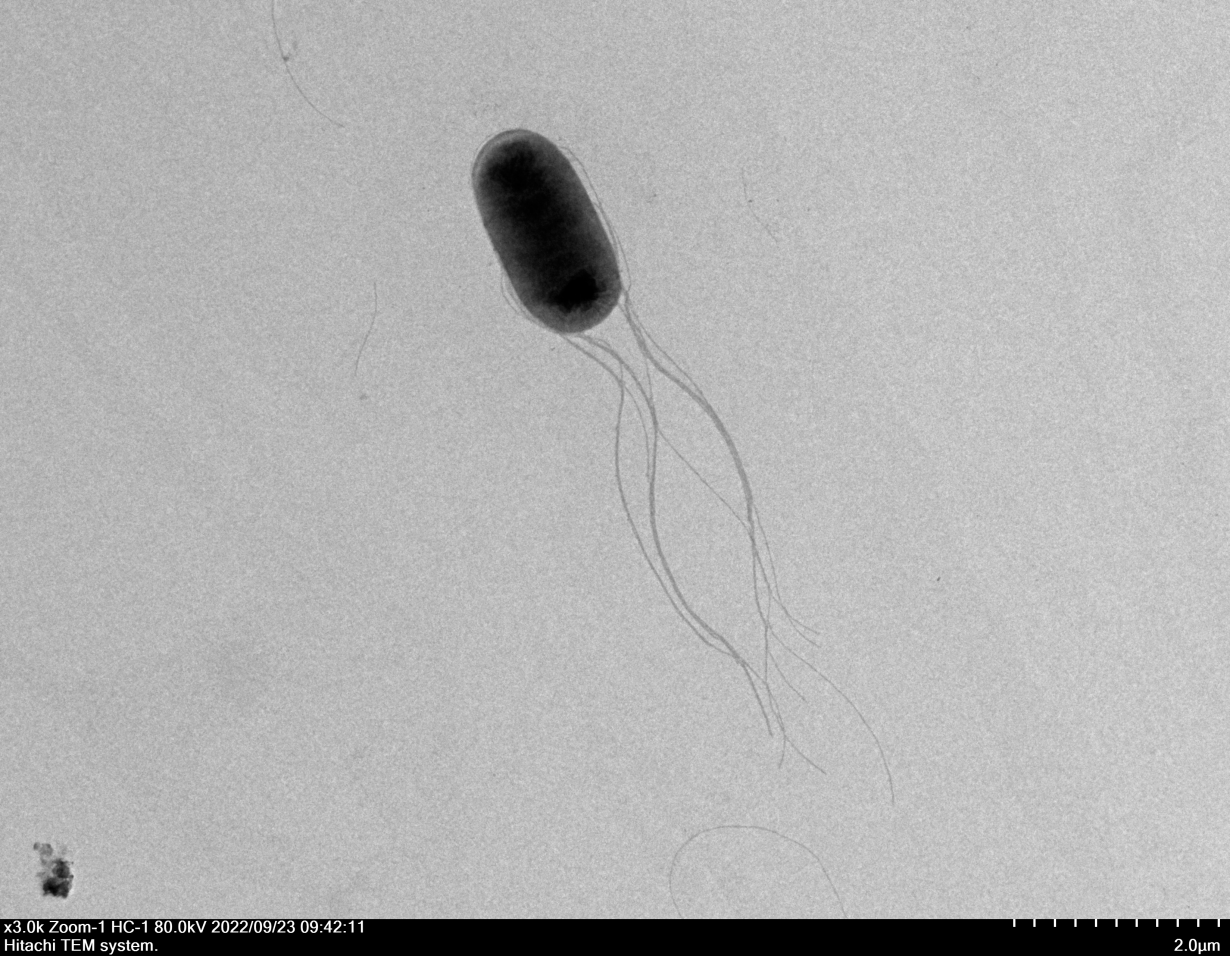
Transmission electron micrograph of strain 21LCBS03^T^. Cells were grown on TSBA at 28 °C for 48 hours and then stained for electron microscopy (HT7700; Hitachi). The scale bar represents 0.5 µm.

**Figure 4:**
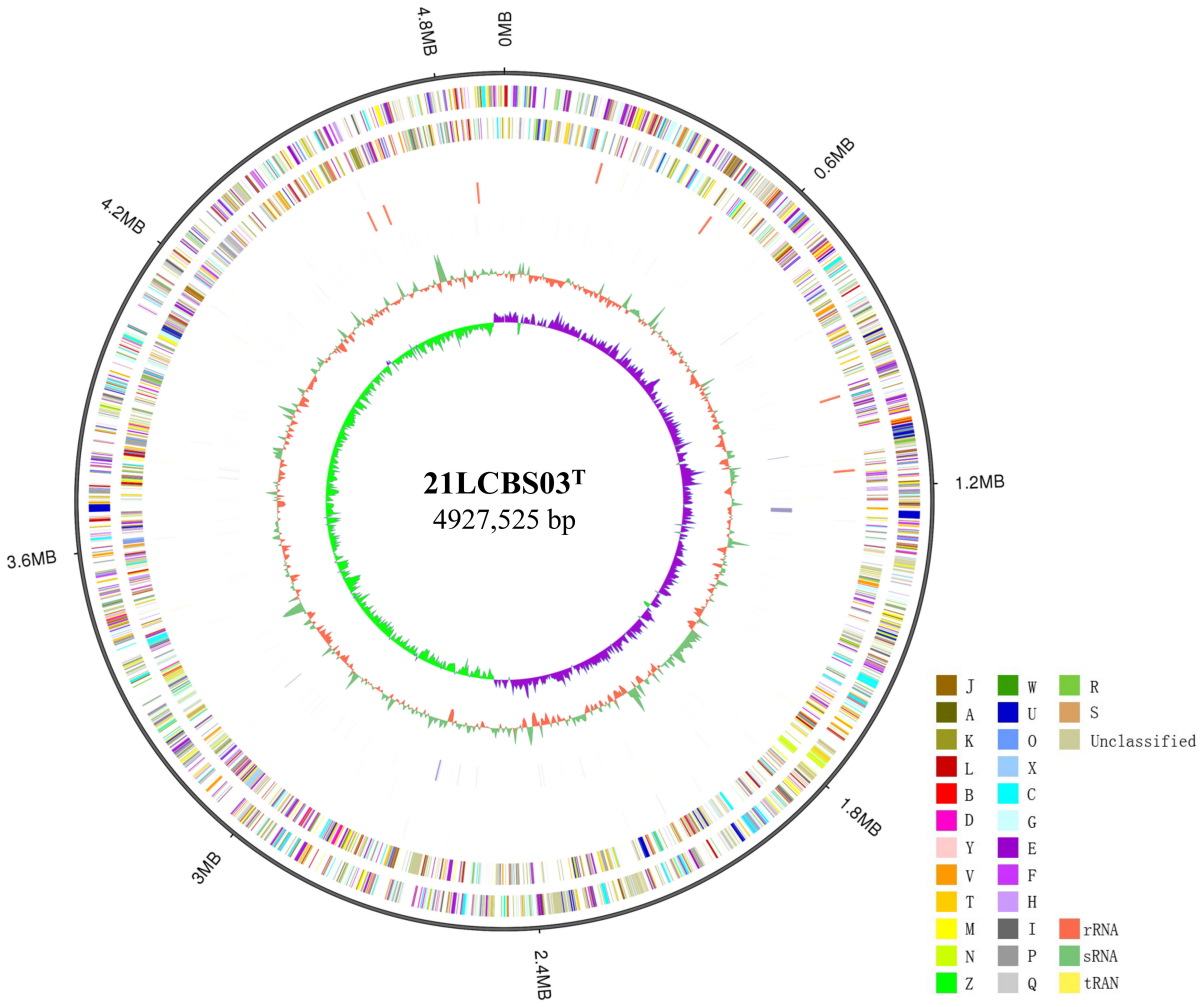
Circular map of the complete genome of *Pectobacterium nicotianae* sp. nov. 21LCBS03^T^. From outside to inside: ring 1, genome size scale; ring 2, forward-strand CDSs colored by COG functional categories; ring 3, reverse-strand CDSs colored by COG categories; ring 4, forward-strand ncRNAs; ring 5, reverse-strand ncRNAs; ring 6, repeat elements; ring 7, G+C content (positive deviation outward in green, negative deviation inward in purple); ring 8, GC skew (positive outward in orange, negative inward in blue).

Genome sequencing of strain 21LCBS03^T^ produced 1314 Mb of raw data (8,764,210 reads, 0.13% duplication) and 1309 Mb of clean data (**Figure 3**). K-mer analysis estimated a genome size of 4.91 Mbp and a depth of 47.51×. GC-depth analysis showed no GC bias, and CheckM2 indicated 100% completeness with 0.44% contamination. The assembled genome was 4,927,525 bp, consisting of a circular chromosome without plasmids, with a GC content of 52.13 mol%, coverage length of 4,927,379, 100% coverage, 270× depth, and 98.96% read usage. The genome contained 4,404 genes: 4,256 protein-coding sequences, 41 pseudogenes, 77 tRNA genes, 1 tmRNA gene, 4 ncRNAs, 29 sRNAs, and 22 rRNAs (8 5S, 16S, and 23S rRNA) (**Figure 4**). Genomic profiles of the other four tobacco isolates were similar and are summarized in **Table 4**.

**Table 4:** Genomic characteristics of five tobacco isolates and the closely related type strains *P. brasiliense* IPO 3540^T^ and *P. jejuense* 13-115^T^.

Strain	21LCBS03^T^	21LCBS02	BSHS4	DL6-4	DL10-2-1	*P. brasiliense* IPO 3540^T^	*P. jejuense* 13-115^T^
Genome size (bp)	4,927,525	4,811,088	4,699,090	4,863,020	4,862,262	4,880,585	5,069,478
Coverage (×)	493	213	251	243	212	40	81
Contig(s)	1	29	49	29	27	7	73
G+C (mol%)	52.13	52.10	52.08	52.14	52.14	52.16	52.03
Total genes	4,404	4,320	4,297	4,315	4,312	4,364	4,506
Protein coding genes	4,256	4,169	4,140	4,155	4,154	4,159	4,360
Pseudogenes	41	59	68	67	66	92	63
tRNA genes	77	68	69	70	70	81	71
tmRNA gene	1	1	1	1	1	1	1
ncRNA genes	4	2	2	2	2	3	2
rRNA genes	22	18	14	17	16	25	6
Completeness (%)	100	100	100	100	100	100	100
Contamination (%)	0.44	0.55	0.41	0.41	0.41	1.28	0.39

### Genomic comparison

**Figure 5:**
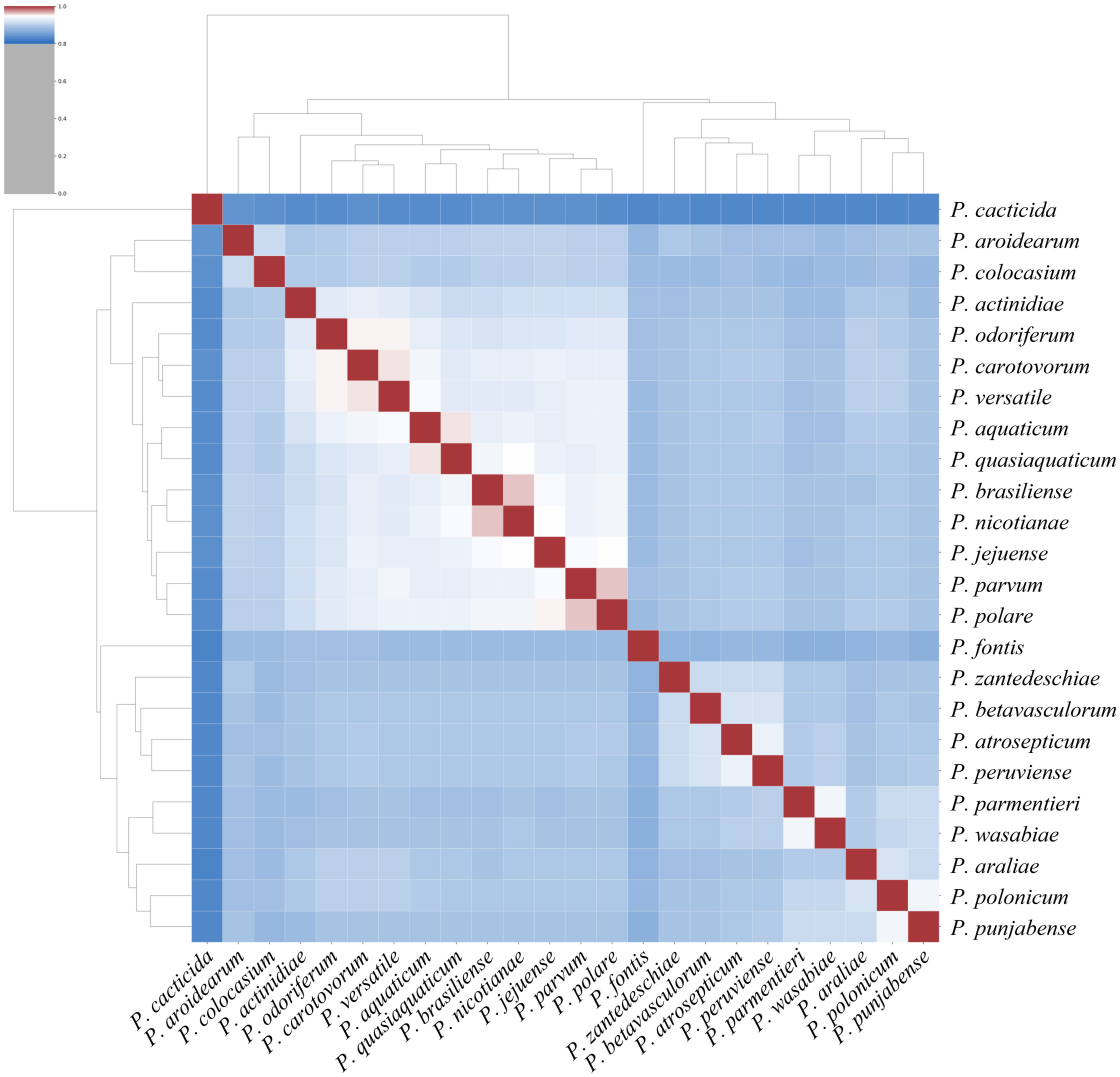
ANIb identity heatmap comparing tobacco strain 21LCBS03^T^ with Pectobacterium type strains. Genome sequences of Pectobacterium type strains used in the analysis are listed in **Table 1**. ANIb identities were computed with pyANI-plus v1.0.0 using the default settings (Pritchard et al., 2016).

To calculate ANI values, the genome sequences of five tobacco strains were compared with those of 23 closely related *Pectobacterium* type strains using the BLAST algorithm (ANIb, the standard value) (Goris et al., 2007; Richter and Rosselló-Móra, 2009; Yoon et al., 2017) and the rapid alignment tool MUMmer (ANIm) via the JSpecies WS v4.3.2 web server (Richter et al., 2016; Lindsey et al., 2023). Data showed that ANI values exceeded 95% when comparing several closely related type strains. The ANI values (ANIb 95.81% and ANIm 95.20%) approached the 95–96% ANI threshold between *P. aquaticum* A212-S19-A16^T^ and the recently proposed novel type strain of *P. quasiaquaticum* A477-S1-J17^T^ (Pédron et al., 2019; Ben Moussa et al., 2021). Additional ANI values exceeded 95% using MUMmer, including ANIm values of 95.69% and 95.24% when comparing *P. carotovorum* DSM 30168^T^ with *P. versatile* CFBP 6051^T^ or *P. odoriferum* NCPPB 3839^T^, and an ANIm value of 95.07% for *P. aquaticum* A212-S19-A16^T^ and *P. polare* NIBIO 1006^T^ (**Supplementary Table 2**). The ANIb values between 21LCBS03^T^ and the other four tobacco strains ranged from 96.96% to 97.63%, with corresponding ANIm values from 97.75% to 97.78%. Strain BSHS4 showed the lowest ANIb and ANIm values, at 96.96% and 97.37%, respectively. Among *Pectobacterium* type strains, *P. brasiliense* IPO 3540^T^ shared the highest ANIb of 95.89% and ANIm of 96.26% compared to 21LCBS03^T^, followed by *P. jejuense* 13-115^T^ (ANIb 94.52%, ANIm 94.91%) and *P. quasiaquaticum* A477-S1-J17^T^ (ANIb 94.02%, ANIm 94.73%). ANI values between 21LCBS03^T^ and other type strains were below 94.0% (**Table 5** and **Figure 5**). These data indicate that five tobacco isolates are at the species cut-off boundary of the 95–96% ANI threshold relative to *P. brasiliense* IPO 3540^T^ (Riesco and Trujillo, 2024).

**Table 5:** Genomic similarity between strain 21LCBS03^T^ and other tobacco isolates or closely related *Pectobacterium* type strains based on dDDH and ANI.

Genome	dDDH^a^			ANIb^b^		ANIm^b^		G+C difference^a^
	dDDH1	dDDH2	dDDH3	ANIb	Aligned	ANIm	Aligned	
DL10-2-1	88.4	80.1	89.8	97.63	88.69	97.78	90.51	0.01
DL6-4	88.4	80.1	89.8	97.63	88.65	97.78	90.51	0.01
21LCBS02	91.4	79.4	92.1	97.49	89.95	97.75	91.65	0.04
BSHS4	82.4	76.2	84.3	96.96	83.79	97.37	85.73	0.05
*P. brasiliense* IPO 3540^T^	79.9	67.9	80.4	95.89	83.92	96.26	85.75	0.03
*P. jejuense* 13-115^T^	79.7	59.3	78.2	94.52	84.75	94.91	87.69	0.10
*P. quasiaquaticum* A477-S1-J17^T^	68.0	58.2	68.0	94.02	73.78	94.73	76.42	0.42
*P. polare* NIBIO 1006^T^	80.2	55.9	77.7	93.76	83.44	94.25	85.64	0.10
*P. parvum* s0421^T^	66.4	54.2	65.6	93.26	74.69	93.91	77.61	1.09
*P. carotovorum* DSM 30168^T^	80.3	51.8	76.4	92.99	82.79	93.45	85.28	0.23
*P. aquaticum* A212-S19-A16^T^	61.3	53.0	60.8	92.87	69.90	93.68	72.52	0.88
*P. versatile* CFBP 6051^T^	81.7	49.8	76.9	92.62	83.91	93.03	87.30	0.22
*P. odoriferum* NCPPB 3839^T^	67.4	48.2	64.6	92.03	77.59	92.61	81.09	0.49
*P. actinidiae* KKH3^T^	70.1	44.7	65.6	91.16	78.29	91.82	80.61	0.59
*P. aroidearum* NCPPB 929^T^	75.9	41.7	69.0	90.33	81.66	90.93	83.80	0.30
“*P. colocasium*” LJ1^T^	70.0	40.3	63.8	89.91	77.33	90.59	80.08	0.48
*P. peruviense* IFB 5232*T*	77.2	38.2	68.3	89.23	80.36	89.82	83.13	1.04
*P. polonicum* DPMP 315^T^	70.0	38.0	62.7	89.11	77.11	89.83	79.18	0.85
*P. atrosepticum* CFBP 1526^T^	69.2	38.1	62.1	89.10	78.90	89.82	80.97	1.04
*P. betavasculorum* NCPPB 2795^T^	61.9	38.3	56.6	88.89	71.97	89.87	74.03	0.97
*P. araliae* MAFF 302110^T^	61.4	37.8	56.0	88.85	72.11	89.76	73.51	1.04
*P. punjabense* SS95^T^	70.4	37.2	62.5	88.80	76.97	89.56	78.38	1.46
*P. wasabiae* CFBP 3304^T^	61.4	37.5	55.9	88.67	74.98	89.60	76.25	1.58
“*P. zantedeschiae*” 9M	71.0	36.9	62.9	88.67	80.05	89.40	81.98	1.39
*P. parmentieri* RNS 08-42-1A^T^	61.2	36.6	55.4	88.42	74.44	89.35	75.51	1.73
*P. fontis* M022^T^	56.8	35.3	51.4	87.74	66.23	88.88	66.90	0.91
*P. cacticida* CFBP 3628^T^	39.6	27.2	35.6	82.99	59.45	85.59	48.24	1.16
*Dickeya solani* IPO 2222^T^	18.0	21.5	17.7	75.33	51.89	84.28	12.65	4.11

^a^ The dDDH values and G + C difference values were yielded using the Genome-to-Genome Distance Calculator (Meier-Kolthoff et al., 2022). DDH1 = formula 1. DDH2 = formula 2. DDH3 = formula 3.

^b^ The ANI (ANIb and ANIm) values were generated using the online service JSpeciesWS (Richter et al., 2016).

The dDDH values were also tested between 21LCBS03^T^ and the other four tobacco strains, as well as the type strains of the genus *Pectobacterium*. Considering error ratios at 70% DDH, dDDH values yielded by the recommended formula 2 (dDDH2), which has a lower error ratio, were chosen for analysis (Auch et al., 2010a, Auch et al., 2010b). Data revealed that all dDDH2 values were less than 70.0% when comparing the genome sequences of each type strain with those of others, with the highest value of 67.8% between *P. polare* NIBIO 1006^T^ and *P. parvum* s0421^T^. Compared with strain 21LCBS03^T^, the four tobacco strains showed dDDH2 values ranging from 76.2% to 80.1%. The lowest value, 76.2% for BSHS4, is below the proposed subspecies threshold of 79.0% but above the species cutoff of 70.0% (Meier-Kolthoff et al., 2014; Riesco and Trujillo, 2024), indicating that this strain could be a new subspecies within the species represented by 21LCBS03^T^. *P. brasiliense* IPO 3540^T^ had a dDDH2 value of 67.9% with strain 21LCBS03^T^, which is below the 70.0% threshold used to define species (Riesco and Trujillo, 2024). The remaining 22 type strains of the genus *Pectobacterium* showed dDDH2 values relative to 21LCBS03^T^ of less than 60.0% (**Table 6** and **Supplementary Table 3**).

**Table 6:** Pairwise ANIb and dDDH2 values between strain 21LCBS03^T^ and closely related type or reference strains.

Strain	ANIb																								
	1	2	3	4	5	6	7	8	9	10	11	12	13	14	15	16	17	18	19	20	21	22	23	24	25
1	–	95.9	94.5	94.0	93.8	93.3	92.9	93.0	92.6	92.0	91.2	90.3	89.9	89.2	89.1	89.1	88.9	88.9	88.8	88.7	88.7	88.4	87.7	83.0	75.3
2	67.9	–	94.2	93.3	93.5	93.0	92.6	92.8	92.5	91.5	91.0	90.3	89.7	89.2	89.1	89.1	88.6	88.7	88.8	88.3	88.9	88.3	87.6	83.1	75.3
3	59.3	57.9	–	92.9	94.6	93.8	92.4	93.2	92.8	92.0	91.2	90.2	89.9	89.0	88.9	89.1	88.7	88.8	88.4	88.4	88.6	88.3	87.7	83.0	75.0
4	58.2	55.0	52.9	–	93.2	92.8	95.2	92.6	92.8	91.9	91.0	90.0	89.6	89.1	89.0	89.2	88.8	88.8	88.8	88.4	88.7	88.2	87.7	83.0	75.5
5	55.9	54.6	60.6	53.2	–	96.0	93.0	93.2	93.5	92.5	91.3	89.9	89.8	89.3	89.3	89.2	89.0	89.1	88.7	88.5	88.8	88.3	87.7	82.9	75.3
6	54.2	53.0	57.0	52.3	67.8	–	92.7	93.1	93.5	92.5	91.2	89.9	89.7	89.4	89.4	89.2	88.8	88.9	88.7	88.4	88.8	88.4	87.8	83.0	75.3
7	53.0	51.7	51.5	64.2	53.3	52.4	–	93.7	94.1	92.9	91.6	89.7	89.5	89.3	89.1	89.4	88.8	89.2	88.6	88.3	88.7	88.3	87.8	83.0	75.7
8	51.8	51.0	53.3	50.6	52.3	52.2	55.6	–	95.4	94.8	92.7	89.9	89.9	89.4	89.3	89.8	88.8	89.6	88.6	88.5	88.8	88.3	87.8	82.8	75.0
9	49.8	49.4	51.1	50.9	53.5	54.5	57.7	64.1	–	94.6	92.5	89.6	89.7	89.1	89.1	89.6	88.8	89.6	88.5	88.3	88.7	88.2	87.7	82.8	75.1
10	48.2	47.3	49.0	48.0	49.5	50.0	52.8	61.5	60.8	–	92.3	89.5	89.5	89.2	89.0	89.6	88.7	89.4	88.5	88.0	88.4	88.1	87.9	82.9	75.3
11	44.7	44.0	45.2	44.3	45.2	45.1	46.5	50.9	50.0	50.1	–	89.1	89.2	88.6	88.6	88.7	88.2	88.7	87.8	87.7	88.1	87.6	87.6	82.6	74.9
12	41.3	41.5	40.8	40.2	40.4	40.6	40.3	40.2	39.8	39.3	38.5	–	90.6	88.6	88.4	88.5	88.4	88.1	88.4	88.1	88.9	88.0	87.4	83.4	75.3
13	40.3	39.7	40.6	39.5	40.1	40.0	39.6	40.6	40.0	39.6	39.2	43.3	–	88.0	88.0	88.2	87.6	88.0	87.6	87.5	87.9	87.4	87.5	83.0	75.3
14	38.2	37.9	37.9	37.9	38.3	38.6	38.4	38.5	38.2	38.2	37.0	36.4	35.3	–	93.5	89.2	91.5	88.7	89.2	89.6	91.1	89.4	87.0	82.4	74.8
15	38.1	38.1	37.9	38.0	38.5	38.9	38.4	38.7	38.4	38.4	37.3	36.3	35.5	54.0	–	89.1	91.3	88.6	89.1	89.6	90.8	89.4	87.1	82.3	74.7
16	38.0	37.6	38.0	38.3	38.3	38.2	39.0	40.2	39.6	39.6	37.7	36.4	35.7	38.2	38.2	–	88.5	91.6	93.5	90.5	88.8	90.6	87.1	82.4	74.8
17	38.3	38.2	38.3	37.6	38.3	38.1	37.8	38.0	38.0	37.7	36.8	36.6	35.3	47.3	46.9	37.5	–	88.2	88.7	88.8	90.9	88.5	86.9	82.6	75.0
18	37.8	37.6	37.8	37.7	38.4	38.1	38.8	40.0	39.9	39.8	38.3	35.8	35.6	37.1	37.2	47.1	36.2	–	90.6	89.3	88.3	89.1	87.1	82.1	75.1
19	37.2	37.0	36.7	37.2	37.1	36.8	37.3	36.8	36.4	36.7	35.7	36.5	34.6	38.7	38.4	54.5	37.4	43.8	–	90.8	88.8	91.0	86.7	82.2	74.6
20	37.5	36.5	36.6	36.7	36.8	36.7	36.8	36.8	36.5	36.7	35.8	35.5	34.9	39.9	40.0	42.8	38.5	40.0	43.5	–	88.9	93.7	86.5	82.3	74.9
21	36.9	37.0	36.8	36.9	37.0	37.3	37.2	37.2	37.1	36.7	36.0	37.3	34.9	44.2	43.8	37.2	44.4	36.0	37.3	37.9	–	88.7	86.7	82.4	74.6
22	36.6	36.5	36.3	36.2	36.6	36.6	36.6	36.7	36.4	36.5	35.6	35.6	34.8	39.6	39.4	43.1	37.8	39.9	44.3	54.7	37.6	–	86.5	82.3	75.0
23	35.3	35.0	35.3	34.8	35.2	35.3	35.3	35.7	35.4	35.7	35.4	34.6	35.1	33.8	33.9	33.6	33.4	33.5	32.9	32.9	33.2	32.6	–	82.2	75.2
24	27.2	27.3	27.3	26.9	27.1	27.0	27.0	27.0	26.9	26.9	26.8	27.9	27.1	26.4	26.3	26.4	26.4	26.0	26.2	26.5	26.2	26.2	25.8	–	74.3
25	21.5	21.4	21.3	21.2	21.6	21.4	21.4	20.7	21.0	21.2	21.1	21.7	21.7	21.2	21.2	20.7	21.3	21.3	21.1	21.5	20.9	21.5	21.0	21.2	–
dDDH2																									

Strain: 1, 21LCBS03^T^; 2, *P. brasiliense* IPO 3540^T^; 3, *P. jejuense* 13-115^T^; 4, *P. quasiaquaticum* A477-S1-J17^T^; 5, *P. polare* NIBIO 1006^T^; 6, *P. parvum* s0421^T^; 7, *P. aquaticum* A212-S19-A16^T^; 8, *P. carotovorum* DSM 30168^T^; 9, *P. versatile* CFBP 6051^T^; 10, *P. odoriferum* NCPPB 3839^T^; 11, *P. actinidiae* KKH3^T^; 12, *P. aroidearum* L6; 13, “*P. colocasium*” LJ1; 14, *P. peruviense* IFB 5232^T^; 15, *P. atrosepticum* CFBP 1526^T^; 16, *P. polonicum* DPMP 315^T^; 17, *P. betavasculorum* NCPPB 2795^T^; 18, *P. araliae* MAFF 302110^T^; 19, *P. punjabense* SS95^T^; 20, *P. wasabiae* CFBP 3304^T^; 21, “*P. zantedeschiae*” 9M; 22, *P. parmentieri* RNS 08-42-1A^T^; 23, *P. fontis* M022^T^; 24, *P. cacticida* CFBP 3628^T^; 25, *Dickeya solani* IPO 2222^T^. The ANIb and dDDH2 values were yielded using the JSpeciesWS and GGDC web services with default settings, respectively.

**Figure 6:**
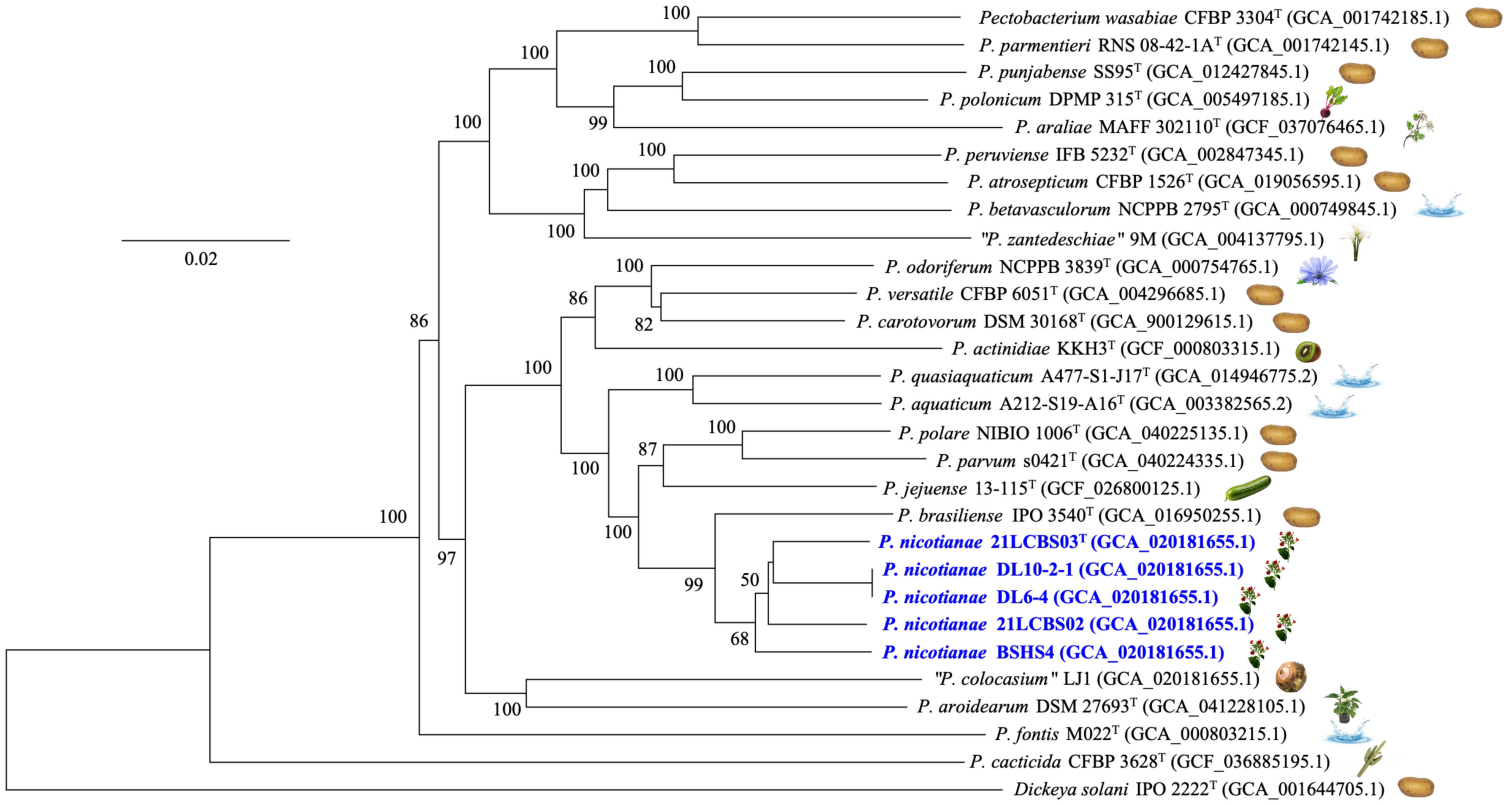
Phylogenomic tree showing the placement of *P. nicotianae* within the genus *Pectobacterium*. The tree was constructed using the Type (Strain) Genome Server (TYGS) and visualized with FigTree v1.4.4. Midpoint rooting was applied. Branch support values (≥50%) from 100 pseudo-bootstrap replicates are shown. Type strains are indicated by superscript T. Sequences obtained in this study are shown in bold blue. *Dickeya solani* IPO 2222^T^ was used as the outgroup. GenBank assembly accession numbers are in parentheses. Colored squares indicate species clusters. Bar, number of substitutions per site.

Phylogenomic analysis showed that all *Pectobacterium* strains formed a large cluster, with at least five main clusters identified (**Figure 6**). The saguaro pathogen *P. cacticida* CFBP 3628^T^ and the water-derived *P. fontis* M022^T^ were the closest to the outgroup *D. solani* IPO 2222^T^. Two monocotyledonous plant pathogens, *P. aroidearum* DSM 27693^T^, isolated from white calla lily, and “*P. colocasium*” LJ1, collected from taro, formed the third cluster. Four potato pathogens, including type strains of *P. parmentieri*, *P. peruviense*, *P. atrosepticum*, and *P. punjabense*, along with other type strains from *P. polonicum* obtained from groundwater in a vegetable field, *P. araliae* causing soft rot of Japanese angelica tree, *P. betavasculorum* obtained from the vascular strand of sugar beet, and “*P. zantedeschiae*” causing soft rot of calla lily, were grouped into the fourth cluster. The fifth cluster was composed of 11 clades, including five potato pathogenic strains of *P. versatile*, *P. carotovorum*, *P. polare*, *P. parvum*, and *P. brasiliense*, two water source strains of *P. quasiaquaticum* and *P. aquaticum*, one chicory pathogenic strain of *P. odoriferum*, one Chinese gooseberry pathogenic bacterium of *P. actinidiae*, and a recently proposed species, *P. jejuense*, causing cucumber stem rot disease in South Korea (Hong et al., 2023). These data showed that ten *Pectobacterium* species can cause soft rot in potatoes, suggesting that the pathogen of potato soft rot is a *Pectobacterium* species complex. This phenomenon might also exist in other plants. Within the fifth cluster, five tobacco strains formed a distinct clade, separated from the closely related type strain *P. brasiliense* IPO 3540^T^ by a branch support value of 99%, suggesting that these strains represent a novel species within the genus *Pectobacterium* (**Figure 6**).

In summary, the five tobacco strains constitute a single species that is distinct from the closest-related species, *P. brasiliense* IPO 3540^T^, and represent a novel plant-pathogenic bacterium. Strain BSHS4 may be a novel subspecies within the species represented by 21LCBS03^T^.

### Taxonomic classification of the genus *Pectobacterium*

To confirm the classification of genome-sequenced *Pectobacterium* strains in the NCBI database, sequence similarities were calculated between 633 strains and the type species, 21LCBS03^T^, using the GGDC and the FastANI script with default settings. Compared with strain 21LCBS03^T^, 121 strains (19.12%), including 109 previously classified *as P. brasiliense*, 10 as *P. carotovorum*, and two unnamed *Pectobacterium* strains, shared dDDH2 values > 70.0% and FastANI values > 96.0%, which exceed the species cut-off values, indicating close relatedness to strain 21LCBS03^T^. However, these strains had dDDH2 values of 63.60~68.80% and FastANI values of 95.27~96.26% with *P. brasiliense* IPO 3540^T^, which were below the 70.0% dDDH2 threshold recommended for species delineation. These data suggest that the 121 strains and 21LCBS03^T^ belong to the same species. Furthermore, 19 strains had dDDH2 values > 70.0% and corresponding FastANI values of 98.19-100.00%, including *P. brasiliense* IPO 3540^T^, suggesting that these 19 strains belong to *P. brasiliense* (**Supplementary Tables 4**, **5**).

Compared with the other 22 type strains, we found that 473 strains had dDDH2 values > 70.0% and FastANI values > 96.0%. The top ten-hit species were *P. versatile*, *P. carotovorum*, *P. parmentieri*, *P. aroidearum*, *P. polare*, *P. odoriferum*, *P. atrosepticum*, *P. parvum*, *P. punjabense*, and *P. actinidiae*, with counts of 120, 72, 63, 44, 31, 25, 24, 15, 12, and 10, respectively.

Several *Pectobacterium* strains may be mislabeled in the NCBI database. Both strains of NBRC 14082 and MAFF 301937 were misidentified as *P. carotovorum* because they shared FastANI values above 98.0% with *P. actinidiae* KKH3^T^. *P. carotovorum* F164 had a FastANI value of 98.52% with *P. aquaticum* A212-S19-A16^T^. Four previously named *P. carotovorum* strains, including PCCS1, PC1, MAFF 301645, and HG-49, were suggested to be new members of *P. aroidearum*. *P. carotovorum* strain MAFF 301296 was proposed as *P. atrosepticum. P. carotovorum* strains Ec-155 and BF20 were renamed *P. brasiliense*. *Pectobacterium* sp. strain F1–1 was classified as “*P. colocasium*”. Strains of *P. polare* IPO 4059 and *Pectobacterium* sp. PL64 were identified as the recently proposed *P. jejuense*. *P. carotovorum* strains NBRC 3830 and MAFF 301877 were verified as *P. odoriferum. P. carotovorum* MAFF 301879 and four *P. polare* strains, WBC9, WBC6, WBC11, and WBC1, were suggested as *P. parvum*. All 30 *P. polare* strains were mislabeled as *P. polaris*. *P. carotovorum* MAFF 311524 should be named *P. polonicum*. Strains of *P. carotovorum* MAFF 301899 and *Pectobacterium* sp. IFB 5596 were suggested as *P. punjabense. P. aquaticum* NAK 467 was renamed as a member of *P. quasiaquaticum. P. atrosepticum* Ec-110 and 14 *P. carotovorum* strains, including RC5297, NBRC 3380, MAFF 730217, MAFF 106664, Ecc15, Ec-180, Ec-179, Ec-177, Ec-168, Ec-093, Ec-008, Ec-007, Ec-006, and 15, were proposed as *P. versatile.*

**Figure 7:**
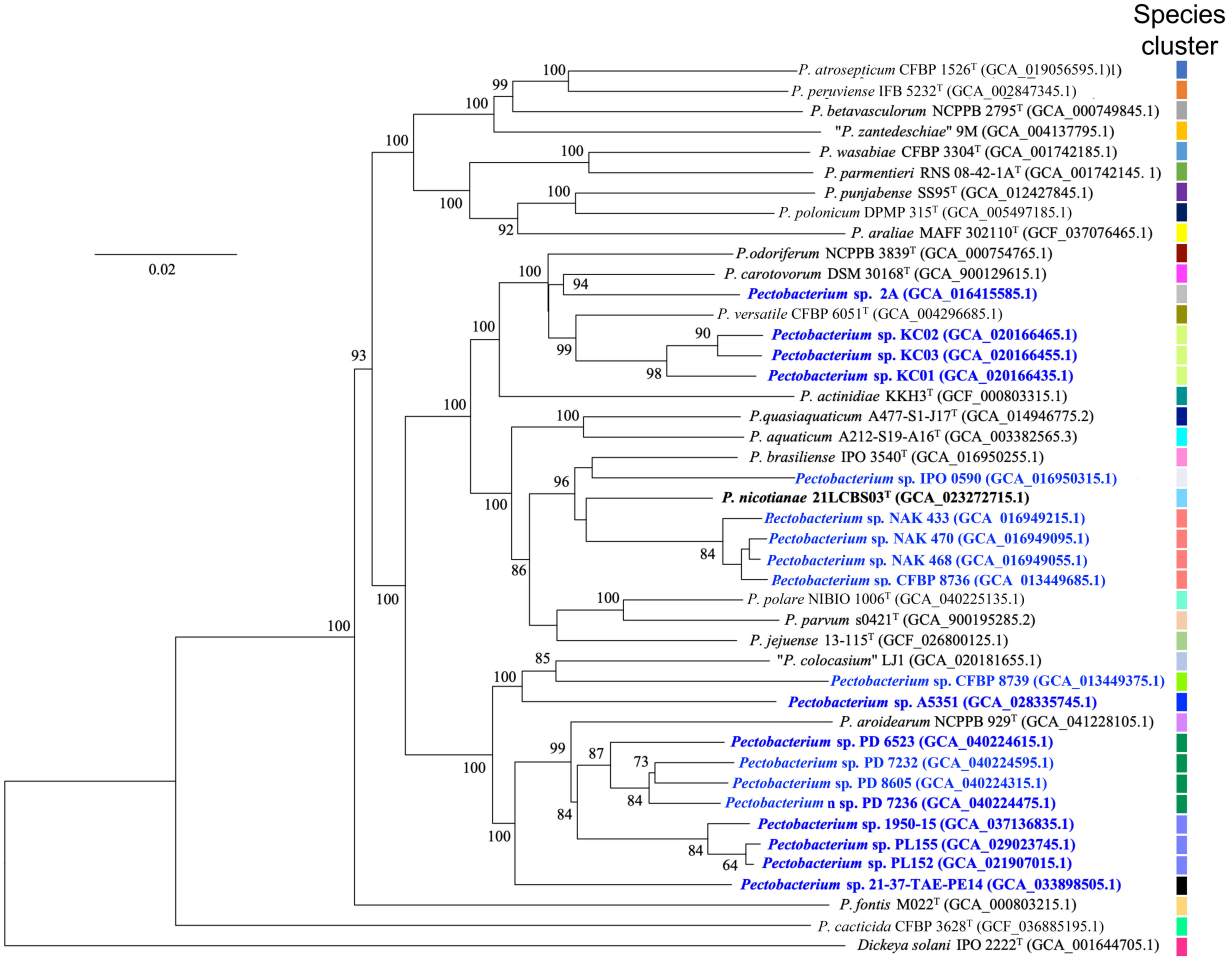
Phylogenomic tree of nine putative novel species within the genus *Pectobacterium*. The tree was constructed using the TYGS web server with default settings. Branch support values (≥50%) from 100 GBDP pseudo-bootstrap replicates are shown (average branch support = 84.7%). Type strains are indicated by superscript T. Putative novel species are shown in bold blue. *Dickeya solani* IPO 2222^T^ was used as the outgroup. GenBank accession numbers are in parentheses—Bar, 0.02 substitutions per nucleotide position. Colored squares indicate distinct species clusters.

Interestingly, data also showed that 19 *Pectobacterium* spp. strains could not be assigned to any existing species and appear to form 9 potential novel species, with dDDH2 values below 70.0% and FastANI values below 96.0% relative to any tested reference or type strain (**Supplementary Table 6**). Phylogenomic analysis indicated that each putative novel species forms a distinct clade within *Pectobacterium* (**Figure 7**). The first potential novel species includes *Pectobacterium* sp. NAK 433, isolated from an insect, and three water-derived strains (CFBP 8736, NAK 468, and NAK 470). The second potential novel species comprises strains PD 8605 from *Spathiphyllum* sp., PD 7236 and PD 7232 from *Sansevieria trifasciata*, and PD 6523 from *Yucca gigantea.* A third group comprises pathogenic strains of Chinese cabbage, including KC01, KC02, and KC03, while a fourth group comprises PL152 (*Colocasia esculenta*), PL155 (*Colocasia esculenta*), and 1950-15 (potato). Additionally, each of the strains A5351 from *Aglaonema* sp., 21-37-TAE-PE14 from *Capsicum annuum*, IPO 0590 and 2A from *Solanum tuberosum*, and CFBP 8739 from river water may represent separate novel species, pending further comprehensive analysis. However, as this assignment is based solely on genome-level thresholds and lacks supporting phenotypic or ecological data, the delineation of these groups as novel species remains provisional.

### Comparative genomics

The genus *Pectobacterium* comprises significant plant pathogens responsible for soft rot diseases worldwide. We conducted a comparative genomic analysis of 24 *Pectobacterium* type and/or representative strains (22 complete and 2 draft genomes) (**Supplementary Table 7**). These strains represent diverse geographic origins and ecological niches, with isolation dates spanning 1959 to 2022. Most strains were isolated from potatoes (n=8) and water environments (n=4). GC content across the 24 strains was largely consistent (50.4 mol%–52.2 mol%), and genome sizes ranged from 4.12 Mbp to 5.28 Mbp. Two strains, *P. fontis* M022^T^ isolated from a waterfall in Malaysia and *P. cacticida* CFBP 3628^T^ obtained from a cactus in the USA, have notably smaller genomes (4.15 and 4.12 Mbp) and fewer coding sequences (3,759 and 3,736) than the average of 4,356 ± 180 CDSs in other strains. Phylogenomic analysis identified *P. fontis* M022^T^ and *P. cacticida* CFBP 3628^T^ as deep-branching species (**Supplementary Figure 11**), with reduced genome sizes and low CDS counts indicating genome streamlining or ongoing degeneration. Notably, *P. cacticida* has recently been reclassified as the type species of the novel genus *Alcorniella* (Jonca et al., 2024).

### Secretion systems

The Sec system, comprising the core components encoded by the gene cluster *secABDEFGY* and by *yidC*, was highly conserved across all 24 strains. Only the accessory genes *hlyA* (present in *P. betavasculorum* CFBP 1520 isolated from sunflower and in *P. parmentieri* RNS 08-42-1A^T^ isolated from potato) and *nmpC* (present only in *P. brasiliense* 1692 isolated from potato) showed strain-specific distribution (**Supplementary Table 8**).

The T2SS (Out system) is the primary secretion pathway for PCWDEs. Fourteen core T2SS components (encoded by the *outBCDEFGHIJKLMOS* cluster) are conserved across all tested strains. However, *outN* is absent in five strains: *P. araliae* MAFF 302110^T^ (from Japanese angelica tree), *P. wasabiae* CFBP 3304^T^ (from wasabi), *P. parmentieri* RNS 08-42-1A^T^ (from potato), *P. fontis* M022^T^ (from a waterfall), and *P. cacticida* CFBP 3628^T^ (from cactus).

The T6SS was the most variable secretion system. It was almost completely absent in the reference strains of three species: *P. parvum* YT22221^T^ (from potato), “*P. colocasium*” LJ1 (from taro), and *P. punjabense* SS95^T^ (from potato). In contrast, it was expanded in *P. odoriferum* BC S7, which carries unique genes including *tssM*, *impA*, *impF*, *KVAR_RS14695*, *tssJ*, *tle1*, *tli1*, *tssB*, *tssC*, *tssF*, *tssG*, and *tssK*.

The gene cluster encoding the *Erwinia amylovora* PAI-2 type T3SSa was intact in *P. parvum* YT22221^T^, isolated from potato in China. However, it was almost completely absent in the remaining 23 strains. This pattern aligns with a previous report (Pasanen et al., 2020) and indicates that most *Pectobacterium* strains rely primarily on T2SS-secreted PCWDEs for pathogenicity. PAI-2 type-related genes, including *sctJ*, *sctN*, *sctS*, *sctT*, and *sctW*, were sporadically present in most strains but absent in six strains: three water-environment strains (*P. quasiaquaticum* A477-S1-J17^T^, *P. aquaticum* A212-S19-A16^T^, and *P. fontis* M022^T^) and three pathogenic bacteria (*P. araliae* MAFF 302110^T^ from Japanese angelica tree, *P. wasabiae* CFBP 3304^T^ from wasabi, and *P. parmentieri* RNS 08-42-1A^T^). In contrast, the flagellum-associated T3SSb-coding genes, including the clusters *flgBCEFGHIJM*, *flhABCD*, and *fliAEFGHIJLMNPQRSZ*, as well as *motAB*, *flmH*, and *nueA*, were highly conserved across all 24 strains.

The T4SS and T9SS were almost completely absent across all strains. However, the Flp/Tad pilus system was highly conserved, except for the absence of *tadB/tadC* in *P. araliae* MAFF 302110^T^ (from Japanese angelica tree). Interestingly, the Type I fimbriae gene cluster *fimABCDEFGIK* was detected in *P. cacticida* CFBP 3628^T^ but was absent in the other 23 *Pectobacterium* strains. T1SS-related genes *dnaK*, *ilpA*, and *htpB* were conserved across all tested strains; however, the genes *mrfC* and *mrfD* were identified only in *P. betavasculorum* CFBP 1520, isolated from sunflower.

### Distribution of PCWDEs

A total of 131 genes encoding PCWDEs were analyzed across 24 bacterial strains. The presence or absence of these genes determines each strain’s potential to function at specific stages of infection (**Supplementary Table 9**).

Genes associated with early infection stages, including regulatory genes (*expI*, *expR*, *exuR*, *gacA*) and genes involved in sensing and motility (*trkA*, *yheL*, *flhD*), were conserved across all strains. The Bcs cluster (*bcsABCEGQRZ*), essential for cellulose biosynthesis and early biofilm formation, was present in most plant-pathogenic strains but absent in *P. quasiaquaticum* A477-S1-J17^T^, *P. aquaticum* A212-S19-A16^T^ (aquatic strains), and *P. cacticida* CFBP 3628^T^ (cactus isolate). This absence likely impairs biofilm formation and plant colonization.

Genes involved in mid-infection processes, particularly those related to pectin degradation and utilization, were widely distributed. All strains possessed the key pectinase genes *pelN*, *pelX*, *paeX*, *paeY*, and *pemA*. Genes involved in pectin processing, including the *bgl*, *kdg*, and *kdu* groups; extracellular pectin lyase genes (*pnl*, *pnlG*, *pnlH*); secretion-related genes (*prtDEF*); protease genes (*ppqL*, *prt1*, *prtA-G*, *prtW*); intracellular rhamnose degradation genes (*rhaABEMR*); pectin transport genes (*togABMNT*); and galacturonate catabolism genes (*uxaABC*), were also broadly present. Several *pel* family members (*pelB*, *pelC*, *pelI*, *pelN*, *pelW*, *pelX*, *pelY*, *pelZ*) were detected in most strains.

Strain-specific differences were primarily observed in genes associated with specialized substrate degradation or late-stage infection functions. The complete polygalacturonase cluster *pehAKNW* was detected only in *P. punjabense* SS95^T^, while *pehN* was present in all strains. This pattern suggests that basal polygalacturonase activity is essential for pathogenicity, whereas the full cluster may facilitate specialized pectin utilization. The galactan degradation cluster *ganABEFG* was absent only in the Japanese strains *P. araliae* MAFF 302110^T^ and *P. wasabiae* CFBP 3304^T^. *ganC* was missing in most strains, including 21LCBS03^T^, and *ganL* was also absent in 21LCBS03^T^; both genes were present together only in *P. brasiliense* 1692.

Among cellulose degradation genes, *celV* was present in all strains. *celS* was identified in three strains: *P. peruviense* A350-S18-N16^T^, *P. wasabiae* CFBP 3304^T^, and *P. parmentieri* RNS 08-42-1A^T^. *celB* was absent in nine strains, including *P. wasabiae* CFBP 3304^T^, but was present in *P. actinidiae* GX-Pa1. For mannose metabolism, *manA*, *manX*, and *manY* were universally present. In contrast, *manB* and *manC*, which encode GDP-mannose synthesis, were detected in only six strains, including *P. nicotianae* 21LCBS03^T,^ and were absent from *P. brasiliense* 1692 and the remaining 17 strains.

The xylan degradation cluster *xylABFGH* was absent from four strains: *P. cacticida* CFBP 3628^T^, *P. actinidiae* GX-Pa1, “*P. colocasium*” LJ1, and *P. fontis* M022^T^. The RG-I lyase gene *rhiE* was detected in only seven strains, suggesting adaptation to specific host cell wall structures. For L-arabinose metabolism, the *araABFGH* cluster was present in all strains except *P. cacticida* CFBP 3628^T^, where it was completely absent. An *araE* homolog was detected only in *P. cacticida* CFBP 3628^T^.

Genes associated with late infection stages showed distinct strain-specific distributions. *pecS* was detected only in *P. aquaticum* A212-S19-A16^T^ and *P. wasabiae* CFBP 3304^T^. *ganR* was unique to *P. wasabiae* CFBP 3304^T^. *P. fontis* M022^T^ acquired *egl* via horizontal transfer from *Ralstonia*. The *yesR* gene was exclusive to *P. cacticida* CFBP 3628^T^. *P4* was detected only in *P. parvum* YT22221. *wcaG* was present only in *P. nicotianae* 21LCBS03^T^, *P. carotovorum* ZM1, and *P. aroidearum* NCPPB 929^T^. *bgxA* was detected only in *P. aroidearum* NCPPB 929^T^, “*P. colocasium*” LJ1, and “*P. zantedeschiae*” CFBP 1357. Additionally, *P. cacticida* CFBP 3628^T^ lacked several gene clusters commonly found in *Pectobacterium*. The two aquatic strains, *P. quasiaquaticum* A477-S1-J17^T^ and *P. aquaticum* A212-S19-A16^T^, showed a similar pattern of gene loss, missing *pemB*, *pehA*, *yesW*, *lasA*, and *hrpW*.

Overall, PCWDE-encoding genes in 24 *Pectobacterium* strains showed conserved early-infection functions and variable late-infection and substrate-degradation profiles. Markedly different PCWDE repertoires distinguished *P. nicotianae* 21LCBS03^T^ and *P. brasiliense* 1692: the former possessed mannose synthesis genes (*manB/manC*) and *wcaG* but lacked *ganC/ganL*, whereas the latter harbored the complete *gan* cluster without *manB/manC*, reflecting their divergent adaptive and pathogenic potentials.

## Discussion

Our study isolated five strains from tobacco hollow stalks in the field in Yunnan Province, which were identified as a novel species, *Pectobacterium nicotianae* sp. nov. Genome-wide relatedness indices (ANIb = 95.89%, dDDH2 = 67.9%) place the boundary between *P. nicotianae* sp. nov. and *P. brasiliense* within the borderline range of widely accepted prokaryotic species thresholds, but the dDDH2 value remains below the boundary. Phylogenomic analysis places the five tobacco groups into a single clade that differs from the *P. brasiliense* type strain IPO 3540^T^, further supporting their status as a novel species. Consistent with the 2024 updated minimal standards for bacterial genome taxonomy (Riesco and Trujillo, 2024), the fixed dDDH cutoff of 70.0% clearly separates the two lineages, while the marginal ANI value mirrors the taxonomic precedent of *P. aquaticum* and *P. quasiaquaticum*, a species pair with analogous borderline ANI values formally validated by LPSN (Pédron et al., 2019; Ben Moussa et al., 2021; Meier-Kolthoff et al., 2022). Beyond genome similarity metrics, stable phenotypic and chemotaxonomic disparities serve as robust differential markers: all *P. nicotianae* strains lack β-glucosidase activity in API ZYM assays and exhibit distinct carbon utilization profiles for L-lactic acid and D-glucuronic acid in Biolog tests. Divergent PCWDE repertoires represent the most striking adaptive distinction: *P. nicotianae* encodes the mannose-synthesis genes *manB/manC* and *wcaG* but lacks the complete galactan-degradation *gan* cluster, a genetic configuration that is fully reversed in *P. brasiliense*, reflecting their divergent host-adaptation strategies toward tobacco and potato, respectively.

Among the five isolates assigned to *P. nicotianae*, strain BSHS4 shows consistent intraspecific phenotypic divergence, including the absence of a hypersensitive response in tobacco, attenuated virulence across multiple crop hosts, and unique resistance to ampicillin and tetracycline. Yet genomic data firmly confirm its conspecific affiliation with the type strain 21LCBS03^T^. The pairwise dDDH2 value of 76.2% between BSHS4 and 21LCBS03^T^ exceeds the 70.0% species demarcation threshold but falls slightly below the 79.0% subspecies reference standard proposed in previous genomic taxonomy research (Meier-Kolthoff et al., 2014), indicating that this strain represents a distinct intraspecific ecotype or a novel subspecies rather than a separate novel species. Phylogenomic clustering tightly groups BSHS4 with the other four tobacco isolates, eliminating the possibility of independent taxonomic status.

*Pectobacterium* strains infect a wide range of plants. At least 11 type strains within this genus were obtained from potato (**Figure 6**). Our multiyear field surveys of 39 symptomatic tobacco samples from major Yunnan tobacco-growing areas confirm that at least 8 *Pectobacterium* species are associated with tobacco hollow stalk, and *P. nicotianae* is the primary cause of tobacco hollow stalk disease (unpublished data), surpassing the previously recorded pathogens *P. carotovorum*, *P. parvum*, and *P. versatile* (Wang et al., 2017; Lin et al., 2020; Lu et al., 2023c). Epidemiological data indicate that the pathogen primarily spreads via rain splash, with severe tissue softening often occurring after tobacco plant top-cutting and the movement of infected stalks into curing barns during harvest. Most *P. nicotianae* isolates exhibit strong host-specific virulence and elicit hypersensitive responses in tobacco, distinguishing them from potato-adapted *Pectobacterium* species that infect a broader range of hosts. This highlights the host-adaptive evolution of this new taxon. Currently, no registered antibacterial chemicals are available for controlling the disease in local production areas, resulting in annual yield losses due to post-harvest soft rot. The detailed taxonomic, pathogenic, and susceptibility data provided by this study address critical gaps in understanding tobacco soft rot pathogens, laying the groundwork for developing species-specific detection methods, field monitoring, and targeted antibacterial strategies to reduce disease losses in Yunnan tobacco fields.

## Taxonomic conclusion

Based on a polyphasic approach integrating phylogenetic, genomic, phenotypic, and chemotaxonomic data, the five tobacco isolates are proposed as a novel species, *Pectobacterium nicotianae* sp. nov. A formal description is provided below.

### Description of *Pectobacterium nicotianae* sp. nov.

*Pectobacterium nicotianae* (ni.co.ti.a’nae. N.L. gen. fem. n. *nicotianae*, of *Nicotiana*, the tobacco plant, from which the type strain (21LCBS03^T^) was isolated).

Cells are Gram-negative, facultatively anaerobic, motile by multiflagella, non-spore-forming, and rod-shaped. Cell dimensions are 0.84-1.10 µm long and 1.34-2.64 µm wide (avg. 0.96 ± 0.08 × 1.90 ± 0.39; *n* = 19). The bacteria can grow on LB, PDA, TSBA, CPG, NA, and TZC agars, with better growth on the first three media. Cells grow at 15-40 °C (optimum, 25-35 °C), at 0-5% NaCl (w/v) (optimum, 0-4%), and at pH 6-10 (optimum, pH 6-9). Cells are catalase-positive and oxidase-negative.

In API 20NE and ZYM analyses, cells are negative for arginine dihydrolase, urease, cytochrome oxidase, esterase lipase (C8), lipase (C14), valine arylamidase, cystine arylamidase, trypsin, α-chymotrypsin, β-glucuronidase, β-glucosidase, N-acetyl-β-glucosaminidase, α-mannosidase, α-fucosidase, and assimilation of maltose, capric acid, adipic acid, and phenylacetic acid; positive for nitrate reduction, glucose fermentation, esculin hydrolysis, β-galactosidase, alkaline phosphatase, acid phosphatase, α-glucosidase, and assimilation of glucose, arabinose, mannose, mannitol, N-acetylglucosamine, malate, and trisodium citrate.

Using GEN III Biolog MicroPlates, *P. nicotianae* strains fail to utilize D-maltose, N-acetyl-D-galactosamine, N-acetyl neuraminic acid, 3-methyl glucose, D-fucose, D-sorbitol, D-arabitol, gelatin, glycyl-L-proline, L-arginine, L-histidine, L-pyroglutamic acid, quinic acid, β-hydroxy-D,L-butyric acid, propionic acid, L-lactic acid, α-hydroxy-butyric acid, α-keto-glutaric acid, and L-fucose; weakly or variably utilize formic acid, glucuronamide, D-glucuronic acid, Tween 40, and acetoacetic acid; and utilize gentiobiose, sucrose, α-D-lactose, D-melibiose, β-methyl-D-glucoside, D-salicin, N-acetyl-D-glucosamine, α-D-glucose, D-mannose, D-fructose, D-galactose, L-rhamnose, D-mannitol, myo-inositol, glycerol, D-glucose-6-PO_4_, D-fructose-6-PO_4_, D-aspartic acid, L-aspartic acid, L-serine, pectin, D-galacturonic acid, L-galactonic acid lactone, D-gluconic acid, mucic acid, D-saccharic acid, L-malic acid, bromo-succinic acid, acetic acid, D-cellobiose, D-trehalose, stachyose, D-raffinose, and citric acid. In the Biolog GENIII assays, the strains are resistant to pH 6, 1% and 4% NaCl, 1% sodium lactate, fusidic acid, rifamycin SV, guanidine HCl, niaproof 4, vancomycin, tetrazolium violet, tetrazolium blue, lincomycin, and lithium chloride; weakly or variably resistant to pH 5, sodium butyrate, and troleandomycin; and susceptible to sodium bromate and potassium tellurite.

The major fatty acids (≥5% of total fatty acids) in the type strain 21LCBS03^T^ are summed feature 3 (C_16:1_ ω7*c* and/or C_16:1_ ω6*c*, C_16:0_), C_16:0_, summed feature 8 (C_18:1_ ω7*c* and/or C_18:1_ ω6*c*), summed feature 2 (C_14:0_ 3-OH/C_16:1_ iso I), and C_12:0_. The polar lipids in strain 21LCBS03^T^ include diphosphatidylglycerol, phosphatidylglycerol, phosphatidylethanolamine, one unidentified aminolipid, two unidentified lipids, and three unidentified aminophospholipids. Only the respiratory quinone MK-8 is present in the type strain.

The type strain, 21LCBS03^T^ (= GDMCC 1.3317^T^ = CCTCC AB 2022131^T^ = JCM 35650^T^), was isolated from a leaf petiole of a tobacco plant (*Nicotiana tabacum*) affected by hollow stalk. The plant was sampled in 2021 in Lincang, Yunnan, China, and the strain is pathogenic to the tobacco variety Yunyan 87. The GenBank accession numbers for the 16S rRNA gene and the genome sequence of 21LCBS03^T^ are ON807178 and CP096821, respectively. The genomic size of the type strain is approximately 4.93 Mbp, and its DNA G+C content is 52.13 mol%.

## Data Availability

The datasets presented in this study can be found in online repositories. The names of the repository/repositories and accession number(s) can be found in the article/**Supplementary Material**.
