# Supplementary material for: Pectobacterium nicotianae sp. nov., a novel pathogen causing tobacco hollow stalk disease, is phylogenetically distinct from P. brasiliense
Source: Front Plant Sci. 2026 Jul 29;17:1895702. doi: 10.3389/fpls.2026.1895702 (PMC13461914; doi:10.3389/fpls.2026.1895702)
Supplement: Supplementary file 1 [file Image1.pdf]

Supplementary Figures

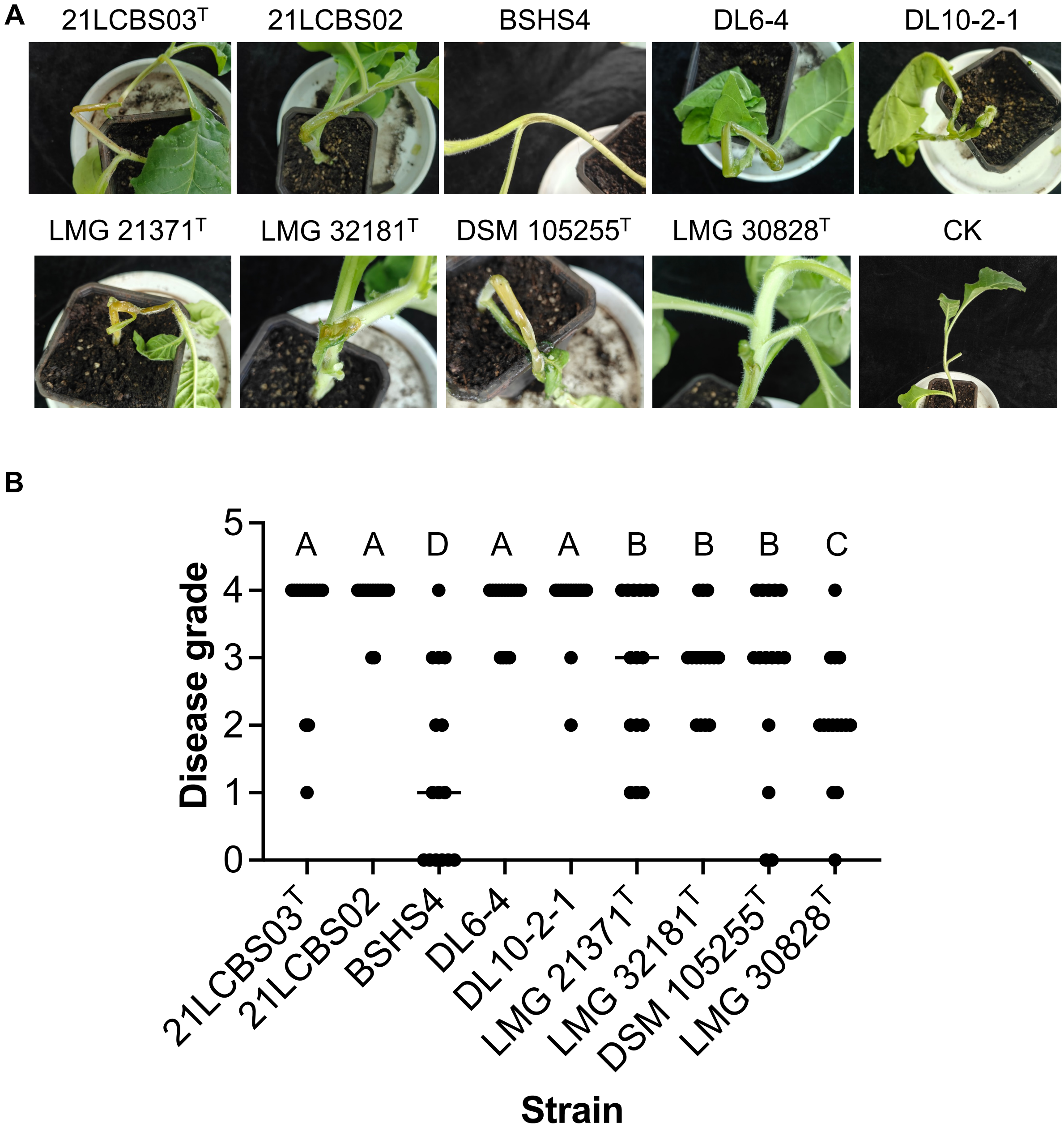

Supplementary Figure 1 **Pathogenicity assessment of diverse bacterial strains via middle-stem injection into 60-day-old *Nicotiana tabacum* cv. Yunyan 87 seedlings at 24 hpi.**

Disease grade for each plant was quantified manually, and all experiments were performed with fifteen biological replicates.

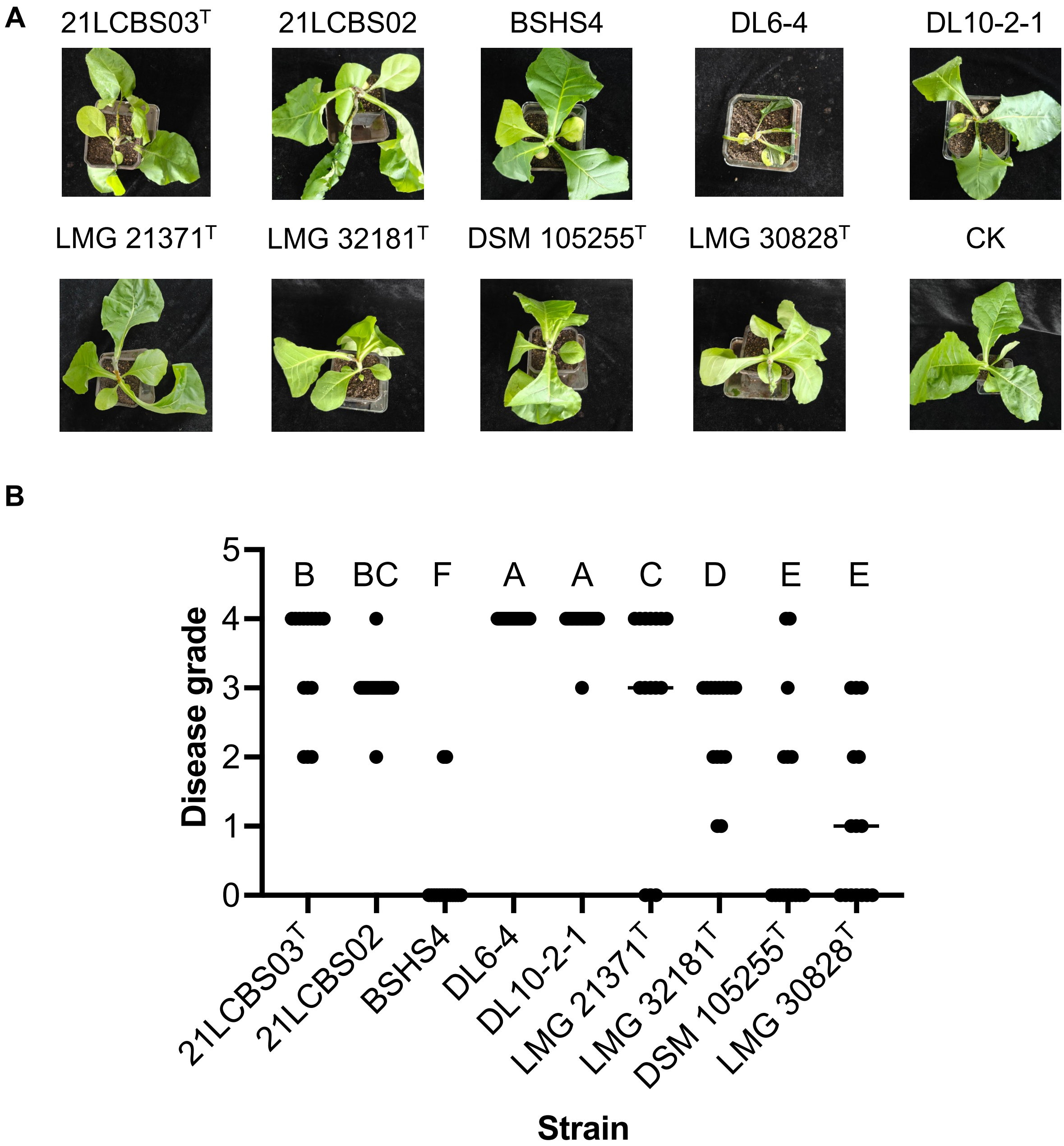

Supplementary Figure 2 **Virulence variation among tested *Pectobacterium* strains on top-cut *Nicotiana tabacum* cv. Yunyan 87 seedlings after 24 hours of surface inoculation.**

Disease grade for each plant was quantified manually, and all experiments were performed with fifteen biological replicates.

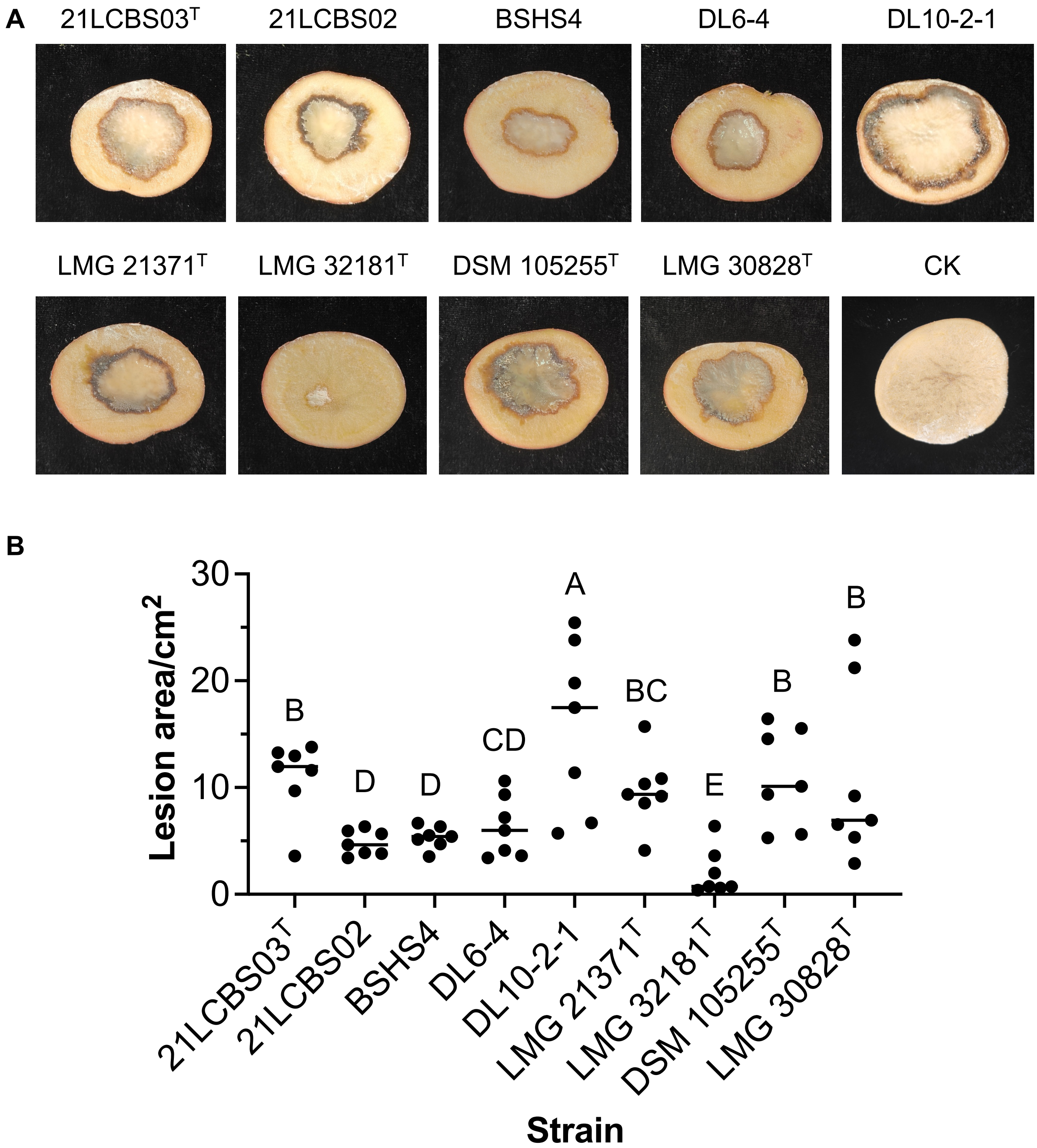

Supplementary Figure 3 Host pathogenicity of different bacterial strains on 1 cm-thick slices of potato tubers at 48 hours post-inoculation.

All lesion areas were quantified using ImageJ 1.53s, and all experiments were performed with seven biological replicates.

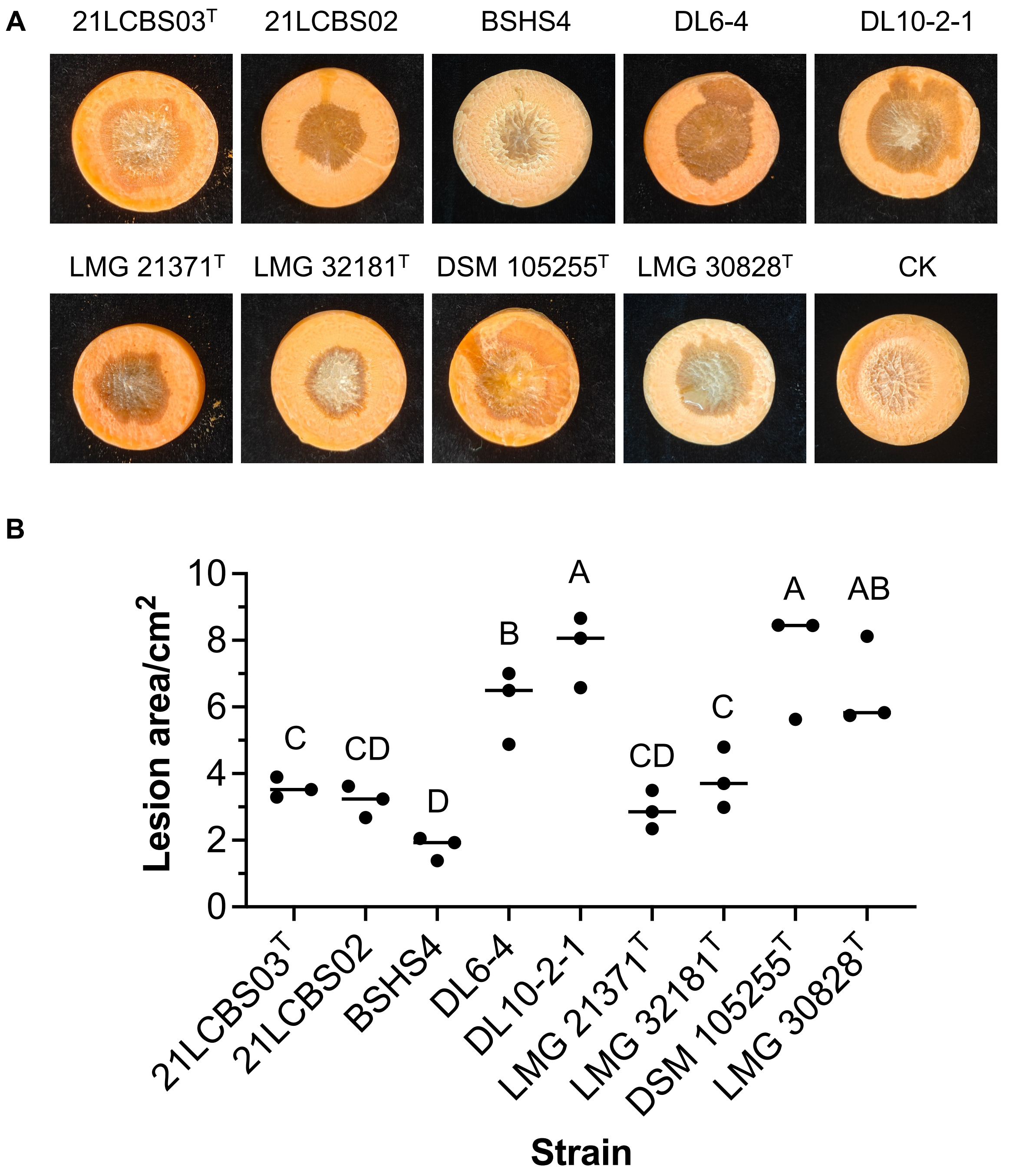

Supplementary Figure 4 Pathogenic characteristics of various tested strains on 1 cm-thick carrot root slices after 48-hour incubation.

All lesion areas were quantified using ImageJ 1.53s, and all experiments were performed in triplicate.

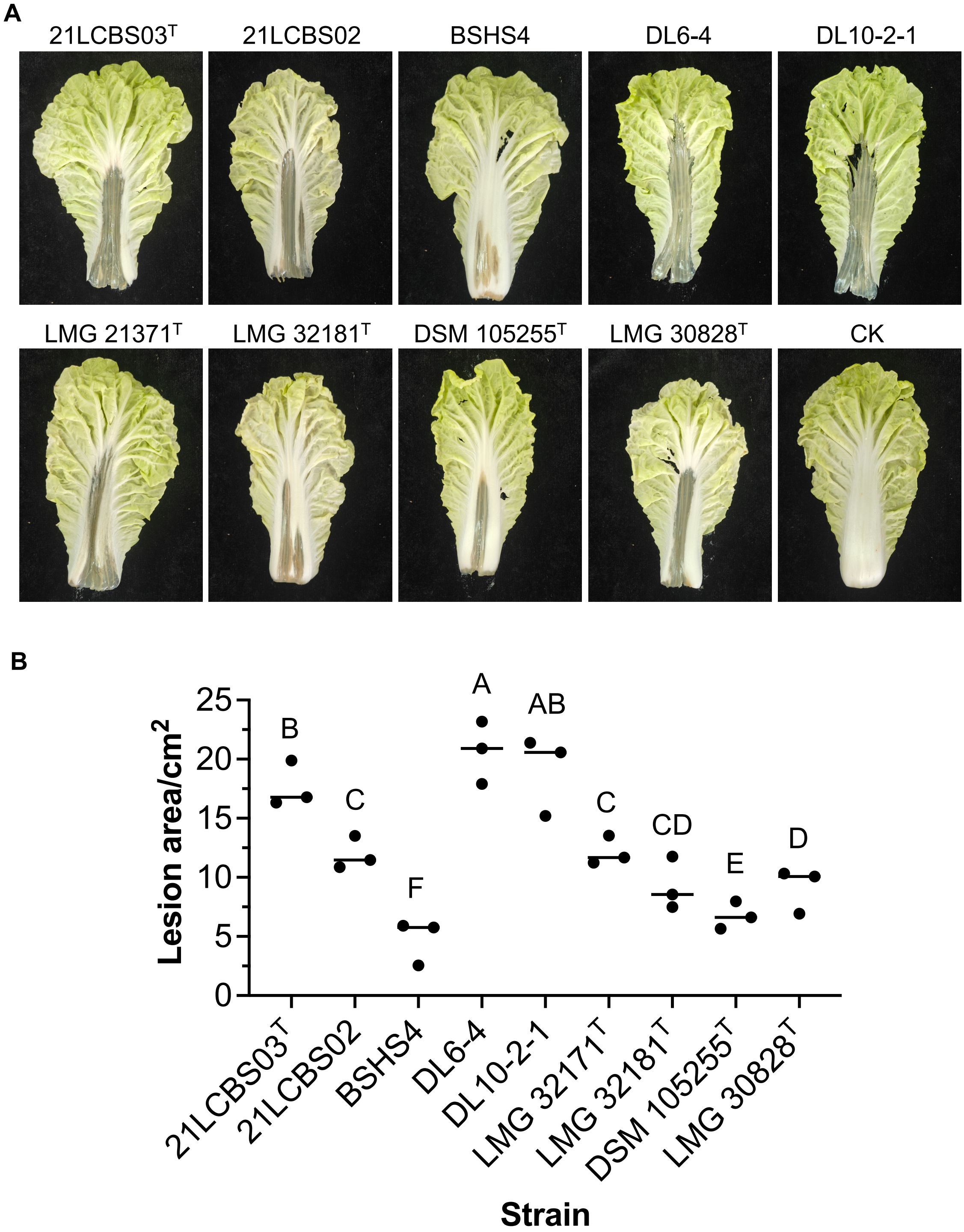

Supplementary Figure 5 Pathogenic performance of tested bacterial strains via stalk injection into Chinese cabbage leaves under 24-hour continuous incubation.

Lesion areas were quantified using ImageJ 1.53s, and all experiments were performed with three biological replicates.

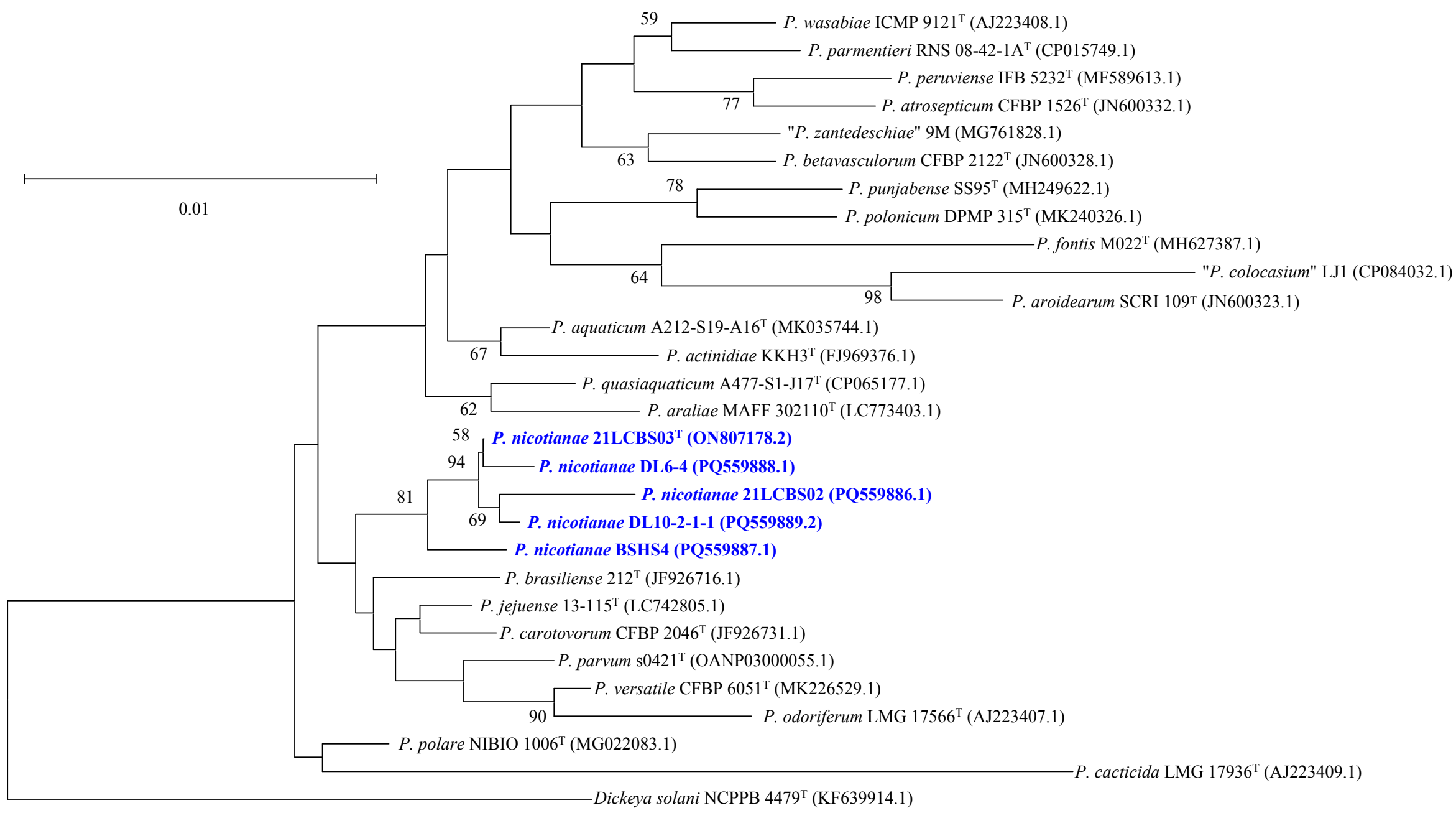

Supplementary Figure 6 Neighbor-joining tree based on 16S rRNA gene sequences, showing the phylogenetic relationships among five tobacco isolates within the genus *Pectobacterium*.

The tree was constructed using MEGA 11 with 1,000 bootstrap replicates (values ≥50% shown). Type strains are indicated by superscript T. Sequences obtained in this study are highlighted in bold blue. *Dickeya solani* NCPPB 4479^T was used as an outgroup. GenBank accession numbers are in parentheses. Bar, 0.01 substitutions per nucleotide position.

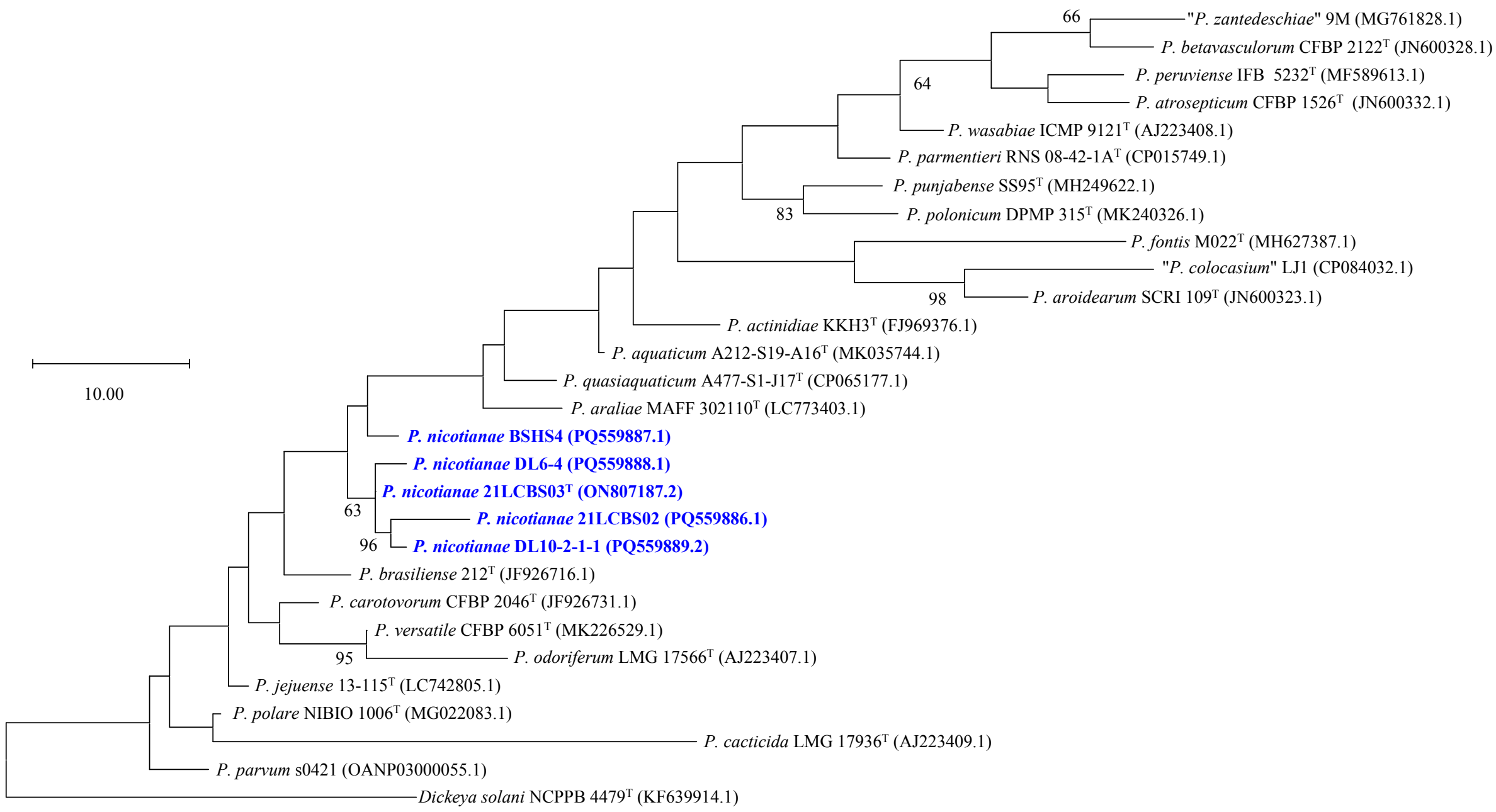

Supplementary Figure 7 Maximum-parsimony tree based on 16S rRNA gene sequences showing the phylogenetic placement of five tobacco isolates.

The tree was constructed in MEGA 11 with 1,000 bootstrap replicates (values ≥50% shown). Type strains are indicated by superscript T. Sequences from this study are shown in bold blue. *Dickeya solani* NCPPB 4479^T served as the outgroup. GenBank accession numbers are in parentheses. Bar, 10 substitutions per nucleotide position.

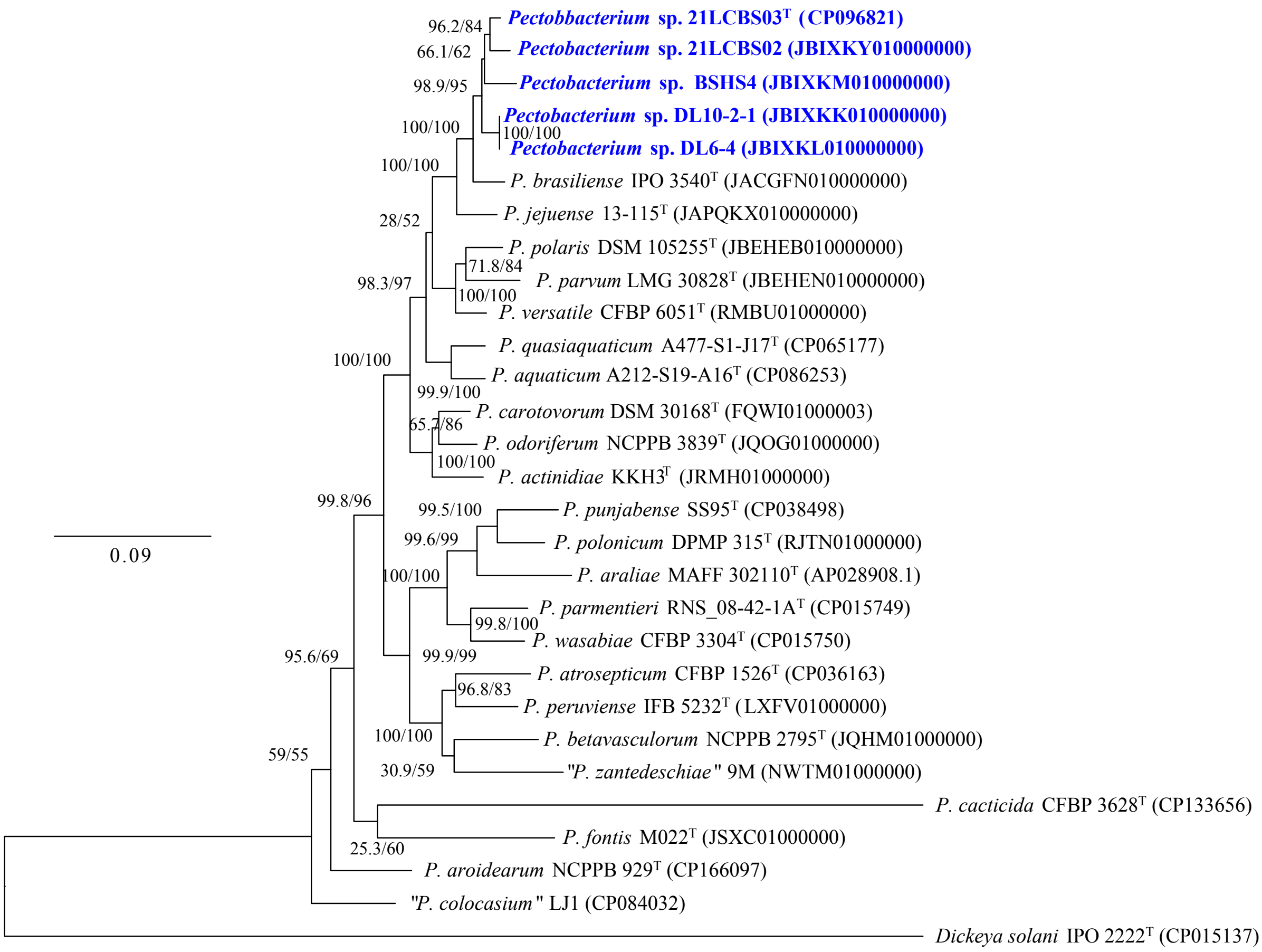

Supplementary Figure 8 Phylogenetic tree based on concatenated DNA sequences of the *dnaX*, *leuS*, and *recA* genes (5,670 bp) showing the relationships among five tobacco isolates.

The tree was constructed in IQ-TREE v2 under the GTR+F+I+R3 model. Node support values are SH-aLRT (left) and ultrafast bootstrap (right), based on 1,000 replicates. Type strains are indicated by superscript T. Sequences from this study are shown in bold blue. *Dickeya solani* IPO 2222^T served as the outgroup. GenBank accession numbers are in parentheses. Bar, 0.09 substitutions per nucleotide position.

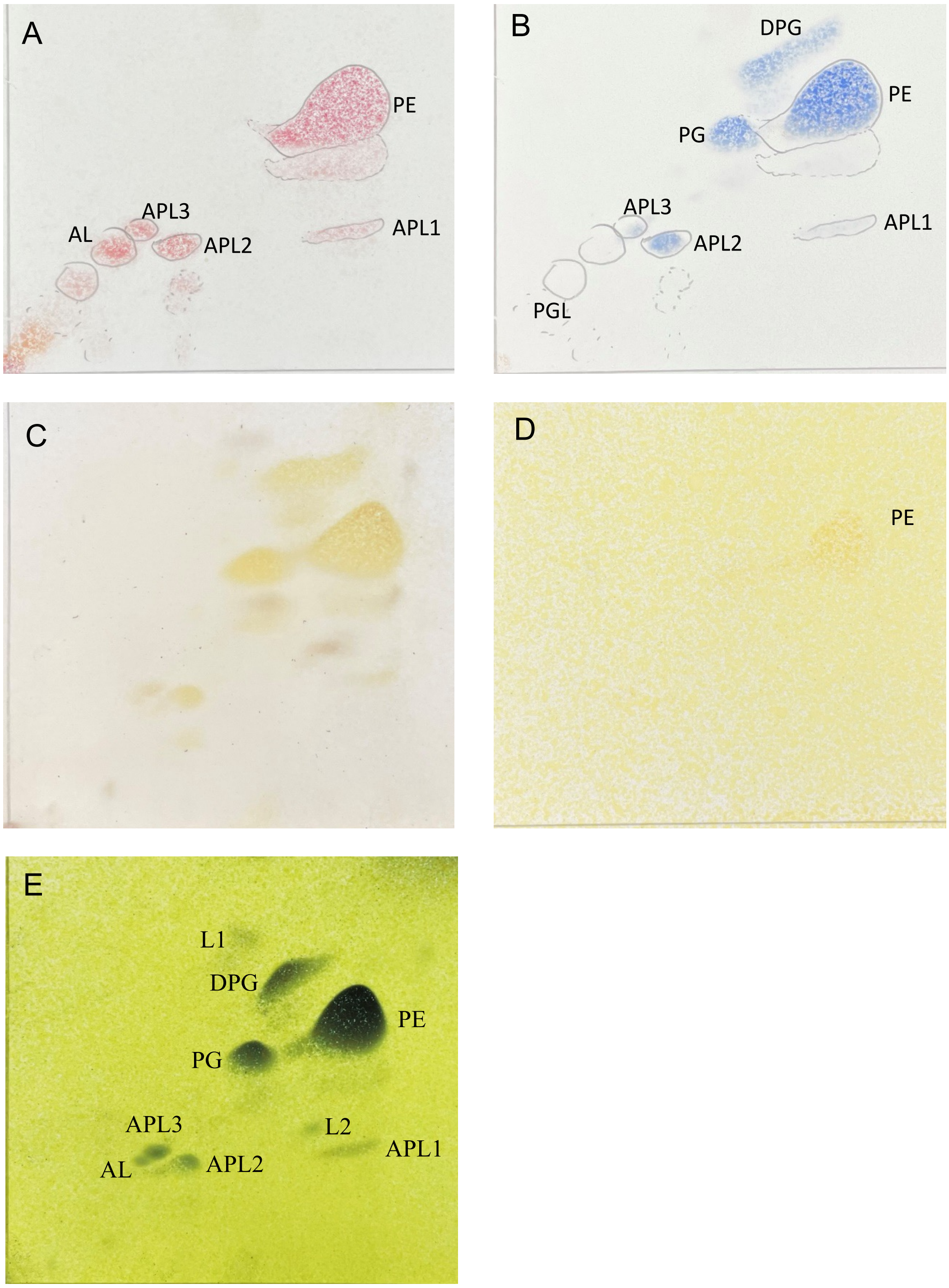

Supplementary Figure 9 Two-dimensional TLC of polar lipids extracted from strain 21LCBS03^{T}.

Lipids were visualized by spraying with: (A) ninhydrin; (B) molybdenum blue; (C) α-naphthol; and (D) 10% molybdophosphoric acid. Abbreviations: DPG, diphosphatidylglycerol; PE, phosphatidylethanolamine; PG, phosphatidylglycerol; L1–L2, unidentified lipids; APL1–APL3, unidentified aminophospholipids; AL, unidentified aminolipid.

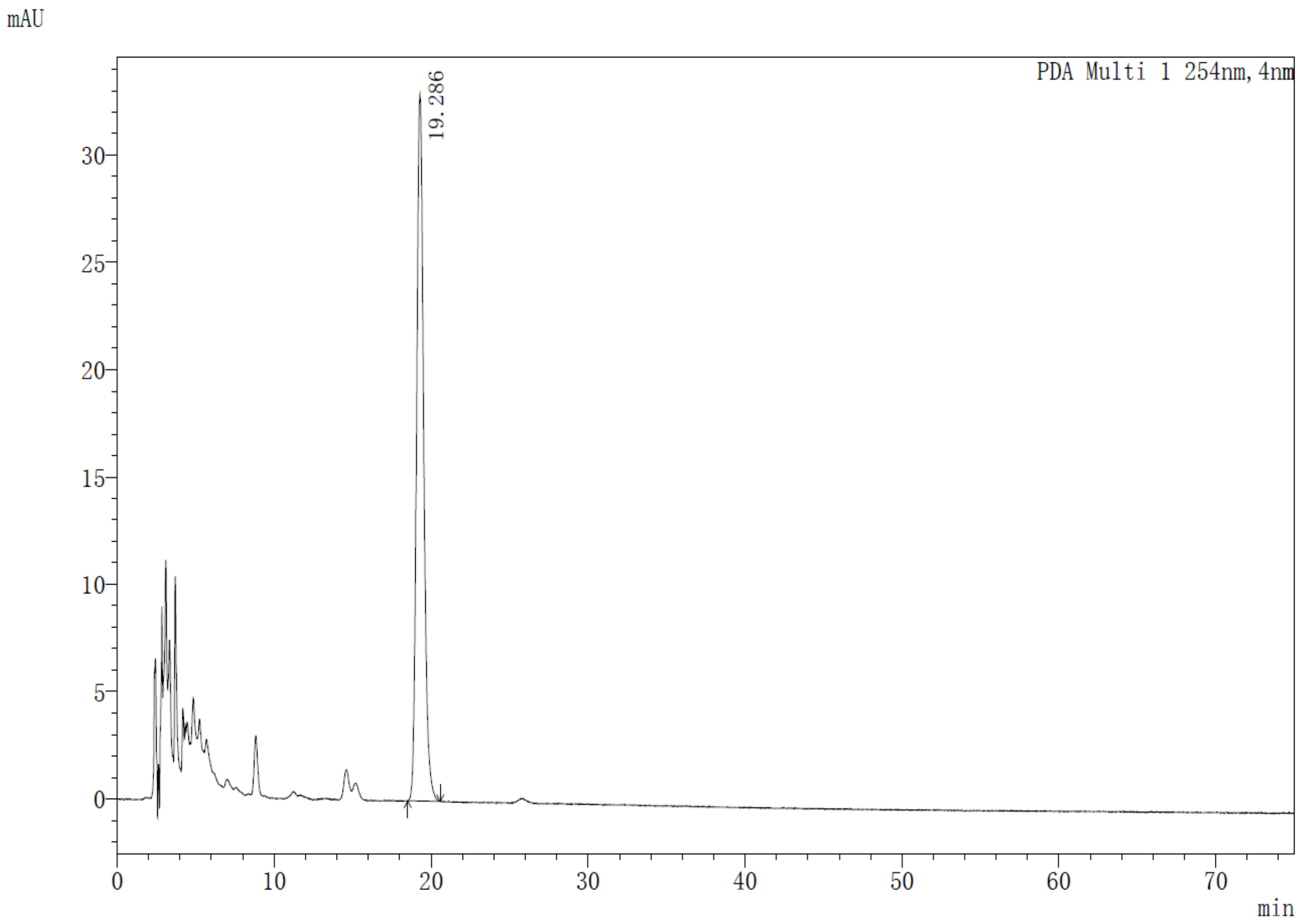

Supplementary Figure 10 High-performance liquid chromatography profile of respiratory quinone MK-8 from strain 21LCBS03^T.

A single peak corresponding to menaquinone MK-8 was detected.

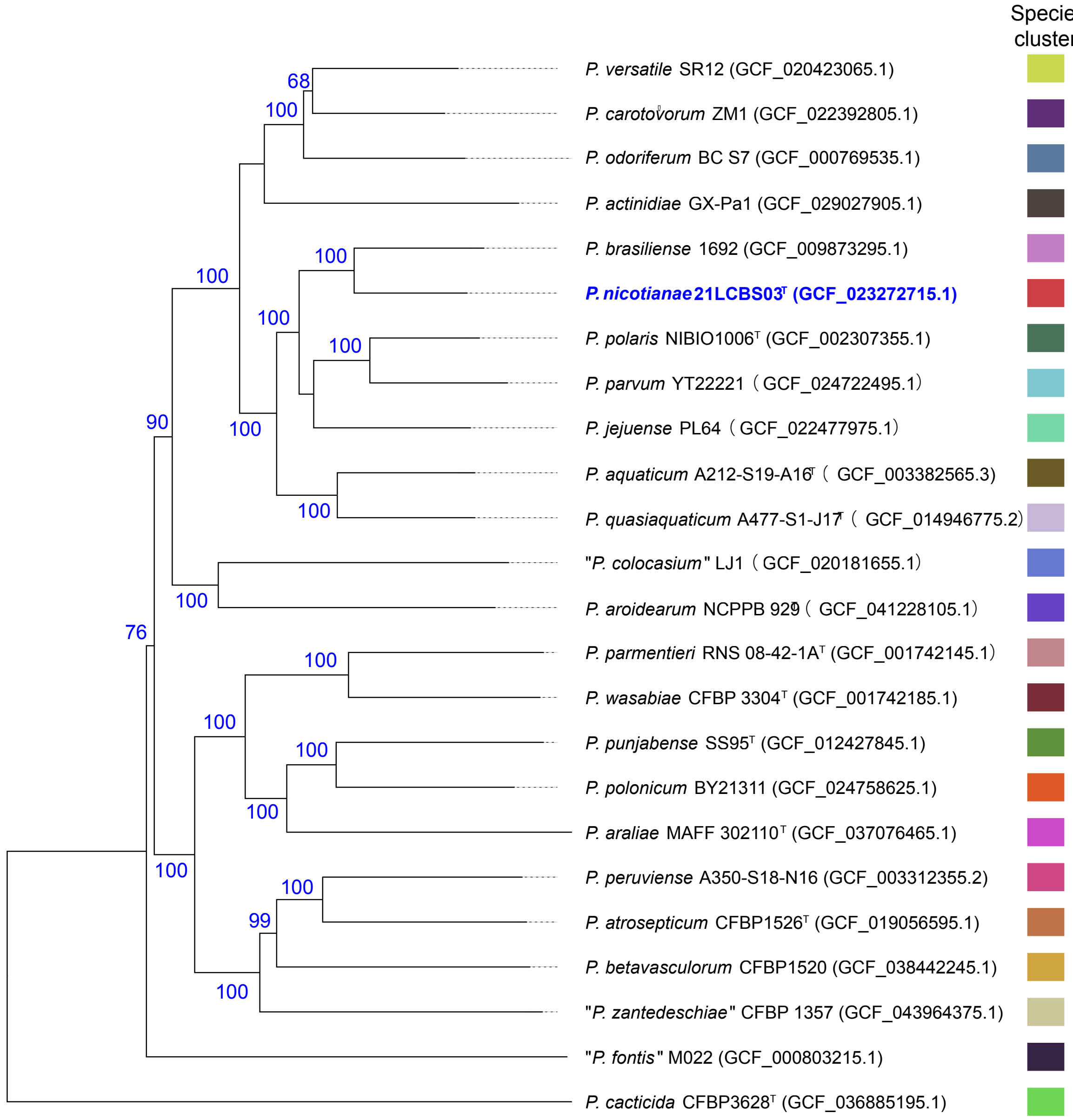

Supplementary Figure 11 Phylogenomic tree showing the relationship between *P. nicotianae* 21LCBS03^T and other type/reference strains of the genus *Pectobacterium*.

The tree was constructed using the TYGS web server. Branch support values (≥50%) from 100 GBDP pseudo-bootstrap replicates are shown (average branch support = 84.7%). The tree was midpoint-rooted. Type strains are indicated by superscript T. Sequences obtained in this study are highlighted in bold blue. GenBank accession numbers are in parentheses. Bar, 0.02 substitutions per nucleotide position. Colored squares indicate distinct species clusters.
